# Emergence of replication timing during early mammalian development

**DOI:** 10.1038/s41586-023-06872-1

**Published:** 2023-12-20

**Authors:** Tsunetoshi Nakatani, Tamas Schauer, Luis Altamirano-Pacheco, Kyle N. Klein, Andreas Ettinger, Mrinmoy Pal, David M. Gilbert, Maria-Elena Torres-Padilla

**Affiliations:** 1 Institute of Epigenetics and Stem Cells, Helmholtz Munich, Munich, Germany; 2 Department of Biological Science, Florida State University, Tallahassee, FL USA; 3 Laboratory of Chromosome Replication and Epigenome Regulation, San Diego Biomedical Research Institute, San Diego, CA USA; 4 Faculty of Biology, Ludwig-Maximilians Universität, Munich, Germany

**Keywords:** Developmental biology, Molecular biology

## Abstract

DNA replication enables genetic inheritance across the kingdoms of life. Replication occurs with a defined temporal order known as the replication timing (RT) programme, leading to organization of the genome into early- or late-replicating regions. RT is cell-type specific, is tightly linked to the three-dimensional nuclear organization of the genome^1,2^ and is considered an epigenetic fingerprint^3^. In spite of its importance in maintaining the epigenome^4^, the developmental regulation of RT in mammals in vivo has not been explored. Here, using single-cell Repli-seq^5^, we generated genome-wide RT maps of mouse embryos from the zygote to the blastocyst stage. Our data show that RT is initially not well defined but becomes defined progressively from the 4-cell stage, coinciding with strengthening of the A and B compartments. We show that transcription contributes to the precision of the RT programme and that the difference in RT between the A and B compartments depends on RNA polymerase II at zygotic genome activation. Our data indicate that the establishment of nuclear organization precedes the acquisition of defined RT features and primes the partitioning of the genome into early- and late-replicating domains. Our work sheds light on the establishment of the epigenome at the beginning of mammalian development and reveals the organizing principles of genome organization.

## Abstract (web summary)

Genome-wide replication timing maps of mouse embryos from the zygote to the blastocyst stage were generated using single-cell Repli-seq, shedding light on the establishment of the epigenome at the beginning of mammalian development.

## Main

Replication timing (RT) is a fundamental epigenetic feature^6^, yet how and when RT is established during mammalian development is unknown. During S phase the genome must replicate once and only once. Replication occurs through a coordinated programme whereby origins of replication fire in a temporally defined order, giving rise to replication patterns characteristic of each cell type^7,8^. Early- and late-replication domains correlate with accessible, actively transcribed euchromatin and silent heterochromatin, respectively^9^. RT is interconnected with other epigenetic features, although their temporal and functional dependency has not been fully established. For example, RT is tightly associated with three-dimensional genome organization, with lamina-associated domains (LADs) and B-type compartments typically corresponding to late-replication domains. Whereas mammalian cells do not possess strongly defined genetic sequences specifying replication origins, replication commences within initiation zones, which are regions of about 40 kb that comprise one or more sites of stochastic origin firing^10,11^. Generally, initiation zones of high efficiency tend to replicate early whereas low-efficiency initiation zones replicate late during S phase. Thus, RT is primarily driven by the probability of initiation within initiation zones. How initiation zones are specified at the beginning of development, and whether cells of the early embryo share a similar structure and features of the RT programme with differentiated cells, remain to be established.

Mammalian development begins with fertilization and is followed by an intense period of chromatin remodelling^12^. Major epigenome features are defined for the first time during this developmental time window: LADs are established de novo in mouse zygotes and the A and B compartments, although detectable in zygotes, gradually become more defined as development progresses towards the blastocyst^13^. Topological-associating domains (TADs) are barely detectable before the 8-cell stage and emerge only at late cleavage stages^14–16^. In mice, zygotic genome activation (ZGA) occurs during this time with minor ZGA occurring in zygotes and the major wave of ZGA in late-2-cell-stage embryos^17^. However, when RT programmes first emerge is unknown. In *Drosophila*, microscopy studies indicate that the onset of late replication emerges after ZGA^18^ but our understanding of this process—and how and when RT is first established in mammals—is unknown.

## RT emerges gradually during preimplantation development

**Fig. 1:**
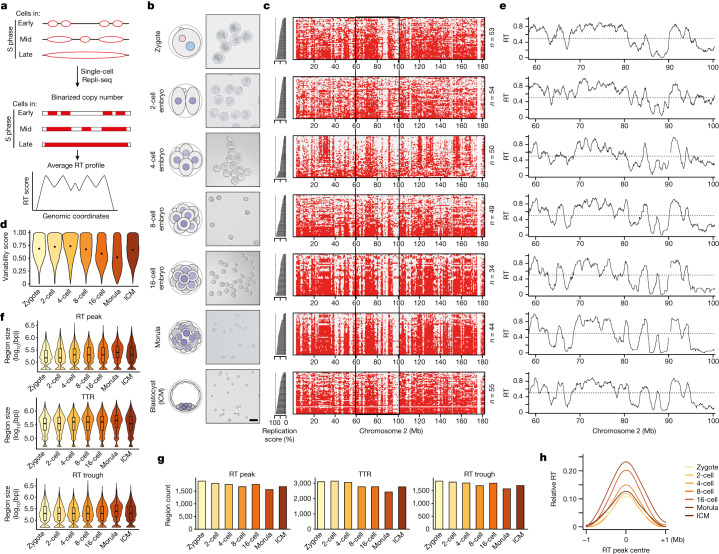
RT emerges gradually during mouse preimplantation development. **a**, Overview of single-cell Repli-seq used to generate RT profiles from single cells in mouse preimplantation embryos based on copy number variation. **b**, Schematic of sampling of embryos and corresponding images of dissociated blastomeres at each stage. The numbers of independent blastomere collections for each stage with similar results are as follows: zygote (3), 2-cell (4), 4-cell (3), 8-cell (3), 16-cell (3), morula (2), ICM (4). Scale bar, 50 μm. **c**, Heatmaps of single cells indicating replication status based on binarized copy number during preimplantation embryogenesis (red, replicated; grey, not replicated). Cells are ranked by their percentage of replicated genome (replication score), which indicates progress in S phase and is plotted as a bar plot on the left. **d**, Variability score during embryonic development; the score is 1 when 50% of cells replicated the genomic bin and 0 when all cells are either replicated (100%) or non-replicated (0%). Each violin plot shows the distribution of scores for all genomic bins. **e**, RT profiles of preimplantation embryos over a representative region on chromosome 2, denoted by black rectangle in **c**. Black line indicates RT profiles, calculated as the average of overlapping intervals defined by genome-wide replication score. **f**,**g**, Size (**f**) and number (**g**) of replication features RT peaks (also known as initiation zones), and RT troughs (also known as termination zones) during preimplantation development. Box plots show median and interquartile range (IQR), and whiskers depict the lowest and highest values within 1.5× IQR. bp, base pair. **h**, Relative RT values centred at RT peaks during embryonic development compared with their neighbouring regions. Note that curves for the 2- and 4-cell stages overlap considerably and, to some extent, with that of zygotes.

**Extended Data Fig. 1:**
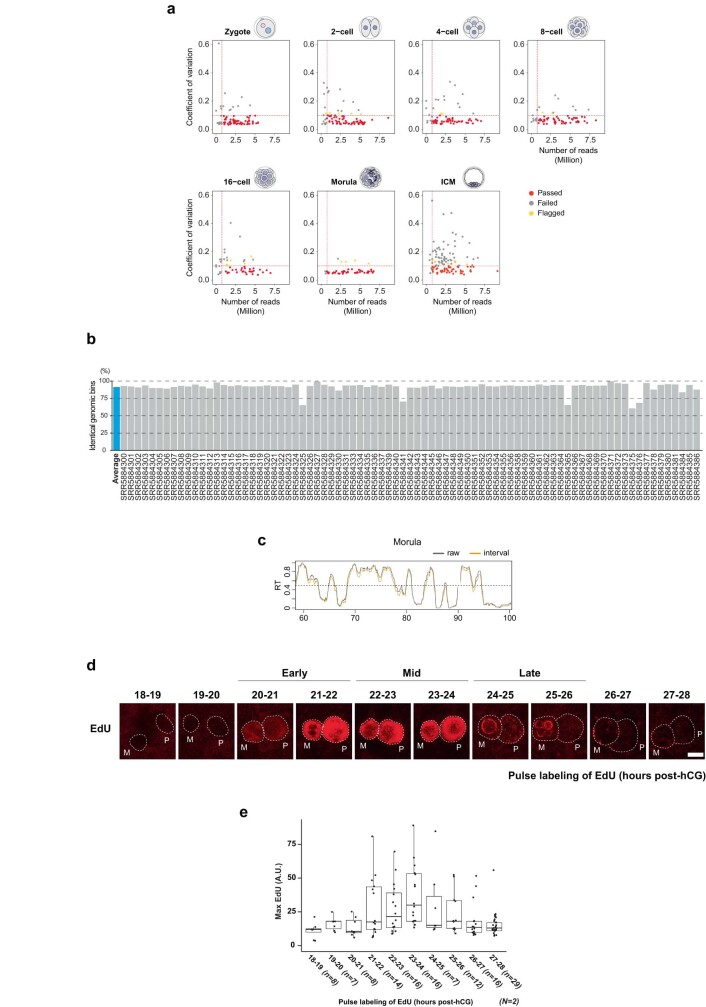
Quality control of scRepli-seq samples. **a**. Scatter plots comparing the coefficient of variation calculated on the average read counts per chromosomes and the number of reads for each cell at the indicated embryonic stages. Horizontal and vertical lines indicate cutoffs for filtering cells. **b**. High concordance of replication state between with or without normalization by cells in G1. **c**. Comparison between two computational methods to calculate RT profiles. Shown are representative RT profiles derived from either raw or interval averaged replication timing values in the morula stage. **d**., **e**. Analysis of DNA replication in zygotes by EdU incorporation (d). Representative images of incorporated EdU and the corresponding quantifications are shown in e. Female and male pronuclei are indicated; the white dotted line depicts the nuclear periphery; note the EdU incorporation at the characteristic ring-shaped heterochromatic regions surrounding the nucleoli precursors between the 24 h and 26 h time. Approximate early, mid, and late S-phase times are indicated based on earlier work. Box plots show median of maximum intensity value and the interquartile range (IQR), whiskers depict the smallest and largest values within 1.5 ×IQR. n, and N, number of analysed embryos and number of independent biological replicates, respectively. Scale bar, 10 μm.

To understand when and how RT emerges during development, we used single-cell Repli-seq^5,19^ in preimplantation mouse embryos (Fig. 1a,b). We collected 529 individual cells of which 53, 54, 50, 49, 34, 44 and 55 passed quality control for zygotes, 2-cell, 4-cell, 8-cell, 16-cell, morula and blastocyst-stage inner cell mass (ICM), respectively (Extended Data Fig. 1a,b, Supplementary Table 1 and Methods). Plotting individual cells based on their replication score, which reflects the percentage of their replicated genome (Fig. 1c), showed a clear replication domain structure consistent with progression of replication, with typical early–late transitions across most stages (Fig. 1c and Extended Data Fig. 1c). Zygotes and 2-cell embryos were an exception and showed a less defined replication pattern across cells and throughout the genome, suggesting a more variable and less coordinated programme (Fig. 1c). This was due to neither absence of DNA synthesis nor embryonic heterogeneity in the progression of DNA synthesis, because we verified microscopically that zygotes showed an expected and consistent spatial pattern of DNA synthesis through S phase (Extended Data Fig. 1d,e). To provide a quantitative metric of the RT programme we computed a variability score, which measures the variance of the replication programme across cells. RT variability score was highest in zygotes and 2-cell and 4-cell embryos but decreased progressively from the 4-cell stage (Fig. 1d). RT of the ICM appeared more variable compared with morula, which may reflect the ICM undergoing cell fate decisions towards epiblast and primitive endoderm^20^, and thus greater heterogeneity in cell identity is likely to be present therein. Overall, the RT programme at the earliest stages of development is less well defined.

**Extended Data Fig. 2:**
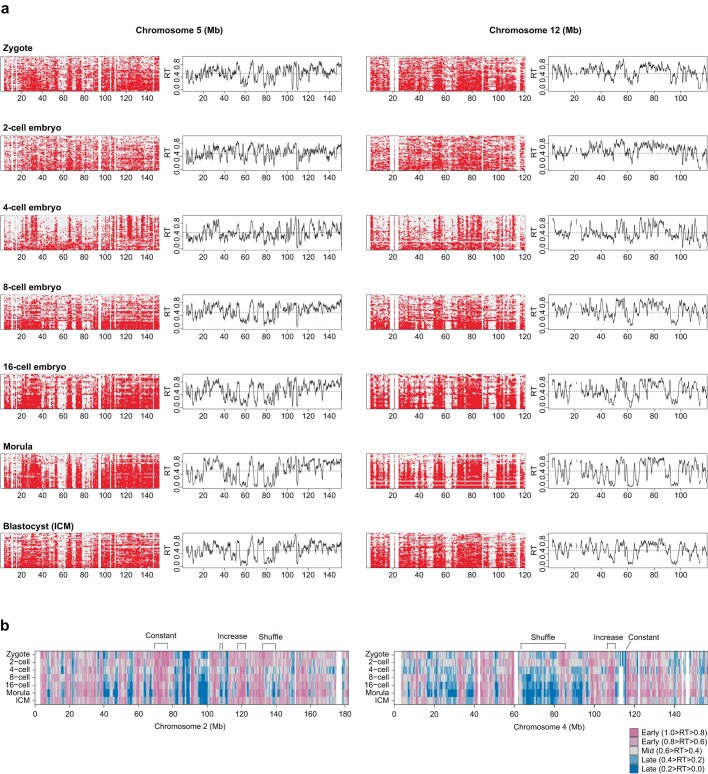
Heatmaps of replication status and RT profiles of preimplantation embryos over a representative region on Chromosome 5 and 12. **a**. Cells are ranked by their replication score. The black line indicates RT profiles calculated as the average of overlapping intervals defined by the genome-wide replication score. **b**. Comparison of RT values in bins of 50 kb across embryonic stages. Representative changes in the RT (increase, shuffle, and constant) are indicated. White regions are regions of no coverage in the corresponding sample.

**Extended Data Fig. 3:**
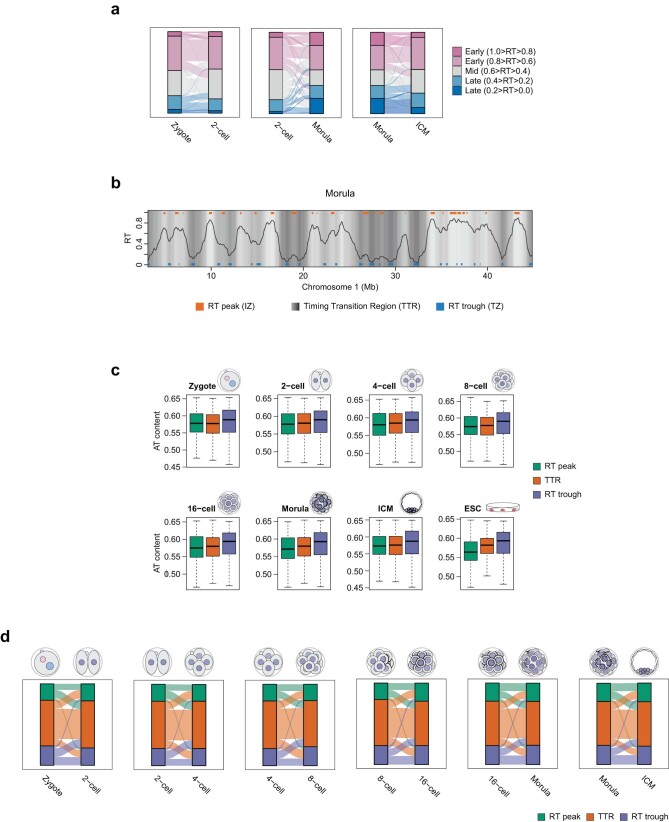
Dynamics of RT peaks, TTRs, and RT troughs during preimplantation development. **a**. Alluvial plot showing the changes of RT phases. RT values were categorised in 5 groups from the earliest (1.0 ≥ RT > 0.8) to latest RT (0.2 > RT ≥ 0.0) across the genome. **b**. Representative replication timing profile in the morula stage depicting RT peaks, TTRs, and RT troughs. Grey shading represents 15 clusters of the RT values that were used to call local maxima (RT peaks or IZs) or local minima (RT troughs or TZs). Regions in transition between RT peaks and RT troughs were called as TTRs. The line indicates RT values. **c**. Fraction of A + T nucleotides in RT peaks, TTRs, and RT troughs during preimplantation development. Box plots show median and the interquartile range (IQR), whiskers depict the smallest and largest values within 1.5 ×IQR. **d**. Alluvial plot showing the relative changes of RT peaks, TTRs, and RT troughs at each cell division during preimplantation development. Box plots show median and the interquartile range (IQR), whiskers depict the smallest and largest values within 1.5 ×IQR.

Embryonic RT profiles showed both early- and late-replication domains, visible as valleys and plateaus (Fig. 1e). Visual inspection showed a progressive delineation of replication domains as development proceeds (Fig. 1e and Extended Data Fig. 2a). This is independent of S-phase length because length is relatively constant until the blastocyst stage^21^. To address whether and how RT changes during development, we compared ‘early’ (RT ≥ 0.5) and ‘late’ (RT ≤ 0.5) RT values from the zygote to the blastocyst ICM. In general, RT values increased towards earlier or later (Extended Data Fig. 2b; increase), indicating definition of the early and late RT programme during development. A portion of the genome showed constant early or late RT throughout (33.1% of the genome replicates early and 16.0% replicates late in all seven stages; Extended Data Fig. 2b; constant). However, some regions shift from early to late RT values and vice versa (Extended Data Fig. 2b; shuffle). For example, 20.9% of the genome switches from early to late RT from 2-cell to morula and 11.1% does so between 8-cell and 16-cell. Likewise, 3.1% changes from late to early RT between 8-cell and morula. This analysis also showed that, whereas some genomic regions do shift RT between early and late values, the most common trend is a progressive definition of RT values towards more early and more late (Extended Data Fig. 2b). Indeed, whereas most of the genome in zygotes and 2-cell embryos (73 and 77%, respectively) shows intermediate RT values (0.4 ≤ RT ≤ 0.8), the genome partitions into RT values spanning the complete S phase as development progresses, resulting in stratification into more extreme early and late RT values after the 2-cell stage (Extended Data Fig. 3a). This behaviour resembles A and B compartments^14^, which undergo progressive increase in compartment strength during cleavage stages^14,16^, suggesting that preimplantation serves as period of gradual establishment of three-dimensional nuclear architecture and RT. We conclude that, although approximately half of the genome preserves its RT, the remaining half undergoes changes in RT as development proceeds and becomes more defined over time.

Next we characterized embryonic RT features by extracting initiation zones, but also zones in which opposing replication forks convene (termination zones) and timing transition regions (TTRs), which are regions located between initiation zones and termination zones^5,7^. Because of the resolution of scRepli-seq. and to distinguish these features from those in methods such as OK-seq and EdU-seq^22,23^, we refer to initiation zones as ‘RT peaks’ and to termination zones as ‘RT troughs’. We defined RT peaks as consecutive bins of local maxima and RT troughs as consecutive bins of local minima of RT values (Extended Data Fig. 3b)^10^. Globally, RT peaks increase in size (*P* = 0.01) with more, smaller RT peaks at early cleavage stages compared with later stages (Fig. 1f,g). Similarly, albeit to a lesser extent, TTRs increase in size (*P* = 0.01; Fig. 1f). The size of RT troughs remains overall stable (*P* = 0.19; Fig. 1f) and, similar to embryonic stem (ES) cells; RT troughs have higher AT content than RT peaks and TTRs (Extended Data Fig. 3c). RT peaks can reshuffle into TTRs and TTRs into RT peaks during each cell division (Extended Data Fig. 3d). Similarly, RT troughs converted into TTRs and TTRs into RT troughs but changes from RT peaks into RT troughs and vice versa are extremely rare (Extended Data Fig. 3d). Approximately half of RT peaks and RT troughs changed into TTRs at the subsequent developmental stage, suggesting remodelling of replication features between each stage following cell division. Because TTRs are regions in which potential changes in RT occur^24,25^, such remodelling may provide the basis for the gradual developmental progression of the RT programme. In addition, the concomitant decrease in the number of RT peaks and their increase in size suggests a progressive consolidation of the RT programme^7^ whereby more adjacent regions with similar RT merge. Indeed, RT peaks become progressively larger and acquire more distinct, earlier relative RT values compared with their genomic surrounding from the 4-cell stage (Fig. 1h). Our data support a gradual consolidation of RT features during preimplantation development and suggest that the shaping of RT occurs at the level of RT peaks and TTRs.

## RT in zygote and 2-cell-stage embryos is distinct from later stages

**Fig. 2:**
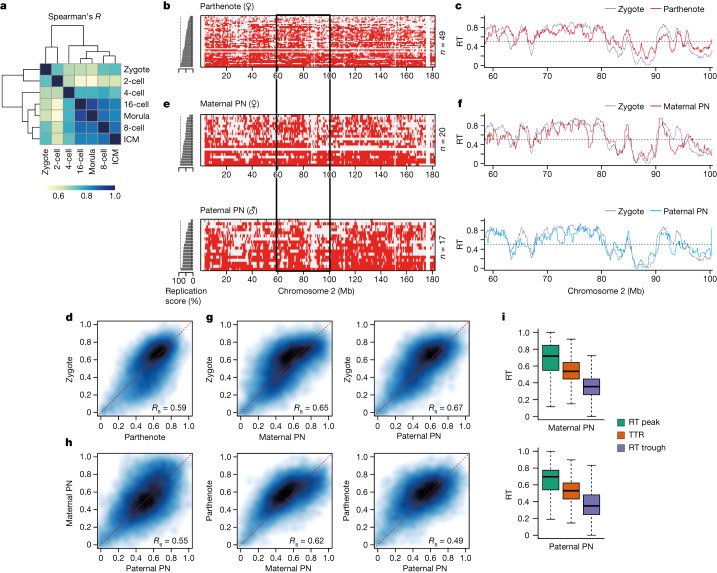
RT in zygotes is less well defined compared with that in later embryonic stages and does not exhibit global allelic differences. **a**, Correlation of genome-wide RT values between the indicated stages of preimplantation embryos using Spearman’s *R*. **b**,**c**, Characterization of RT in parthenogenetic zygotes. Heatmaps show replication states in parthenogenetic embryos (**b**) and corresponding average RT profiles (**c**). **d**, Smoothed scatterplot of RT values in normal versus parthenogenetic zygotes. Spearman’s correlation (*R*_s_) is indicated. **e**,**f**, Characterization of RT in physically isolated maternal and paternal pronuclei (PN). Heatmaps show replication states in each pronucleus (**e**) and corresponding average RT profiles (**f**) of the chromosome 2 region, indicated by the black rectangle. **g**, Smoothed scatterplot of RT values in zygotes compared with isolated maternal and paternal pronuclei. Spearman’s correlation is indicated. **h**, Smoothed scatterplot of RT values comparing maternal or paternal pronucleus and parthenogenetic zygotes, as indicated. Spearman’s correlation is indicated. **i**, RT values of RT peaks, TTRs and RT troughs in isolated maternal and paternal pronuclei. Box plots show median and IQR, whiskers depict the lowest and highest values within 1.5× IQR.

**Extended Data Fig. 4:**
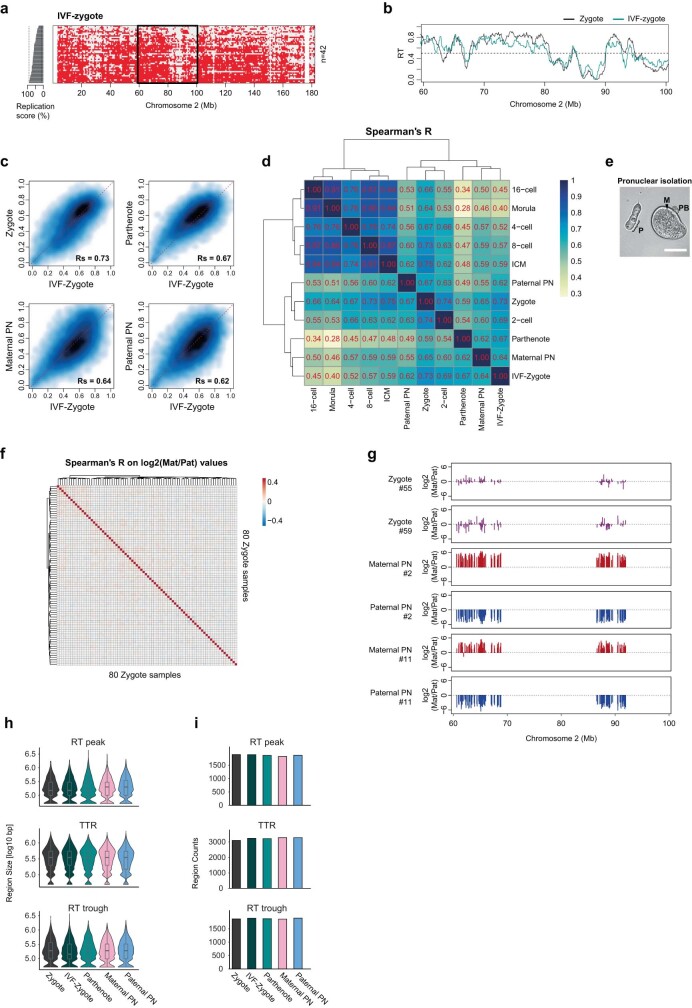
Unique pattern of RT in zygote is not due to differences in replication between maternal and paternal alleles. **a**. Representative heatmap depicting binarized replication status of all single cells in zygotes produced by IVF. Cells are ranked by their percentage of replicated genome (replication score), which is plotted as a bar plot on the left. **b**. Average RT profile of IVF-derived zygotes at the chromosome 2 region indicated by a black rectangle in a. The lines indicate RT profiles calculated as the average of overlapping intervals defined by the genome-wide replication score. **c**. Smoothed scatterplot comparing the RT values in zygotes, parthenogenetic zygotes, and isolated pronuclei (PN) compared to that of IVF-derived zygote. Rs indicate Spearman’s R. **d**. Correlation of genome-wide RT values between normal zygotes, zygotes produced by IVF, parthenogenetic zygotes and isolated maternal and paternal pronucleus (PN) embryos and later developmental stages using Spearman’s R. **e**. Representative brightfield image of isolated paternal pronucleus and remaining maternal pronucleus in the ooplasm. M, P, and PB indicate maternal pronucleus, paternal pronucleus, and polar body, respectively. Pronuclear isolation was repeated twice independently with similar results. Scale bar, 50 μm. **f**. Correlation heatmap of log2 maternal to paternal ratios between individual zygotes after discrimination of parental origins of sequencing reads using SNPs. Allele-specific bias was calculated by computing correlation coefficients of the maternal to paternal ratios across all genomic bins in which SNPs enabled identification of parent-of-origin allele. **g**. Representative genomic tracks of the log2 maternal to paternal ratio in zygotes (magenta) and in physically isolated maternal (red) or paternal pronucleus (blue) samples after assigning parental origin based on SNPs. Regions in which there are no reads (e.g. ~65–85 Mb) correspond to regions with no SNPs. **h**., **i**. Analysis of the size (h) and number (i) of the replication features in normal zygotes compared to zygotes produced by IVF, parthenogenetic zygotes and isolated maternal and paternal pronucleus (PN). Box plots show median and the interquartile range (IQR), whiskers depict the smallest and largest values within 1.5 ×IQR.

**Extended Data Fig. 5:**
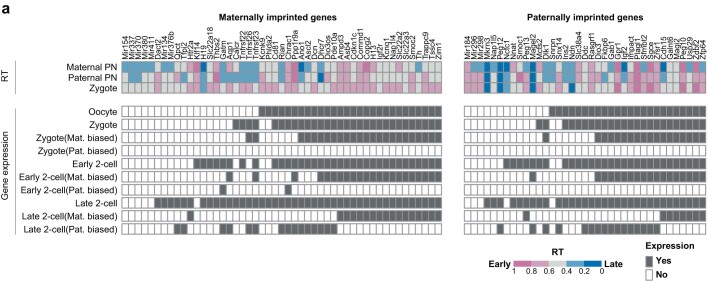
Analysis of imprinted genes indicated no replication asynchrony. **a**. Analysis of RT at maternally (left) or paternally (right) imprinted genes in zygotes and in mechanically isolated paternal and maternal pronuclei (PN). RT values are shown on the top and expression data as ‘yes’ (detected) or ‘no’ (undetectable) is shown on the bottom. Expression data derives from GSE45719 and indicates expression in the indicated stage or maternal and paternal expression bias detected (grey) or undetected/absent (white).

Genome-wide correlation analysis of RT across all stages established that zygotes and 2-cell embryos cluster apart from all other stages (Fig. 2a), suggesting that, despite a similar variability score, the 4-cell-stage RT programme differs from zygotes and 2-cell embryos in other features. To determine the basis of the differences in RT behaviour in zygotes and 2-cell embryos we investigated three alternative explanations. First, to determine whether the unusual RT patterns resulted from asynchrony due to different fertilization times, we performed Repli-seq in zygotes produced by in vitro fertilization (IVF), allowing timely control of fertilization. IVF zygotes showed RT profiles similar to those of zygotes arising from natural fertilization (Extended Data Fig. 4a,b). Second, we considered whether unusual RT patterns result from disparate RT of maternal and paternal genomes, which are thought to replicate asynchronously^26^, are physically separated as two pronuclei during the first cell cycle and remain topologically segregated in 2-cell-stage nuclei^27^. To address this we performed Repli-seq in parthenogenetic zygotes containing only one copy of the maternal genome. The replication profiles in parthenotes and normal zygotes were similar (Fig. 2b,c). Genome-wide correlations of RT values confirmed that RT values in parthenogenetic and naturally fertilized zygotes were comparable, and also with IVF zygotes (Fig. 2d and Extended Data Fig. 4c,d). This analysis confirmed that RT separates into two major groups containing zygotes and 2-cell embryos versus all other stages (Extended Data Fig. 4d). We further generated Repli-seq from physically isolated pronuclei (Extended Data Fig. 4e), which showed overall similar RT profiles in maternal and paternal pronuclei (Fig. 2e,f). Both pronuclei exhibited genome-wide correlations similar to natural zygotes (Spearman’s *R* = 0.65 and 0.67 for maternal and paternal, respectively; Fig. 2g) and to IVF zygotes (Extended Data Fig. 4c). Maternal RT values correlated slightly better with parthenotes than paternal RT values (Spearman’s *R* = 0.62 and 0.49, respectively; Fig. 2h) suggesting that, while highly similar, differences exist between the RT profiles of parental genomes. Finally we investigated whether allele-specific differences can bias RT patterns by performing single-nucleotide polymorphism (SNP)-based analysis of RT in zygotes from hybrid (F_1_ × DBA) crosses. Specifically we asked whether the subtle RT differences between parental genomes are consistent across individual embryos. We find that overall there is no consistent allelic-specific bias in zygotes (Extended Data Fig. 4f,g). This indicates that, although maternal and paternal genomes differ slightly in their RT profiles, these differences do not bias zygotic RT. In agreement, RT peaks, TTRs and RT troughs from both genomes have similar RT behaviour (Fig. 2i and Extended Data Fig. 4h,i). In addition, analysis of imprinted genes indicated no replication asynchrony, in line with findings from ES cells^28^ (Extended Data Fig. 5). We conclude that RT profiles in zygotes are not due to parental asynchrony but rather reflect inherent properties of RT in both genomes at early developmental stages. Therefore, early embryos show a RT programme that is initially less well defined and becomes progressively more defined from the 4-cell stage.

## Segregation between early and late RT increases as development proceeds

**Fig. 3:**
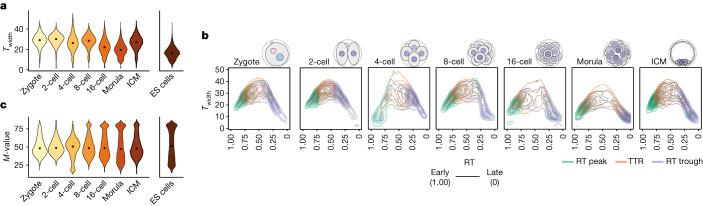
RT heterogeneity decreases with developmental progression, and segregation between early and late RT values increases. **a**, Violin plots showing relative RT heterogeneity (*T*_width_), which is the replication score difference between 25 and 75% of cells replicating the 50 kb bin, in embryonic development and in mouse embryonic stem (ES) cells. Dots indicate median. **b**, Contour plot showing *T*_width_ along progression of RT in mouse embryos. RT peaks, TTRs and RT troughs are indicated. **c**, Violin plots showing RT mid-point value (*M-*value), which is the replication score at which 50% of cells replicated the 50 kb bin during embryonic development and in mouse ES cells. Dots indicate median.

**Extended Data Fig. 6:**
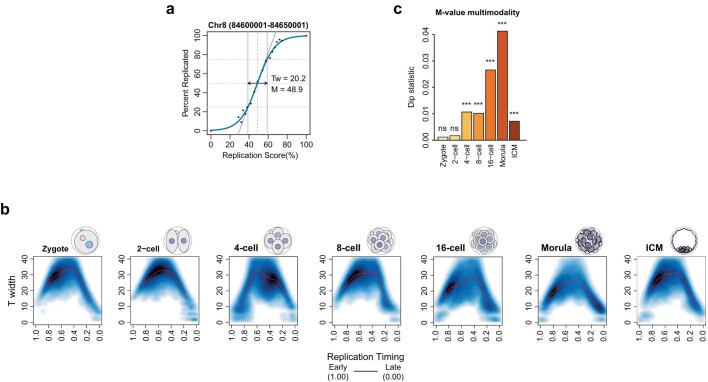
Early and late replicating regions have less heterogeneity than that of mid replicating region. **a**. Representative genomic bin on chromosome 8 depicting the parameters of sigmoid curve fitting. M-value (M) represents the replication score at which 50% of the cells replicated the bin. T width (Tw) was calculated based on replication score difference between 25% and 75% of cells replicated the bin. **b**. Smoothed scatter plots of T width along progression of replication timing for the indicated developmental stages. **c**. Measure for multimodality of M-values during embryonic development by Hartigans’ dip test. A greater dip value suggests a greater deviation from unimodal distribution (at least bimodal). Asterisks indicate significance levels (***, p-value < 0.001; ns, non-significant, as follows: for zygotes 9.8e − 01; for 2-cell 5.7e − 01; for 4-cell <2.2e − 16; for 8-cell <2.2e − 16; for morula <2.2e − 16; for ICM < 2.2e − 16).

Next, we investigated whether the robustness of RT (cell-to-cell heterogeneity) changes during development. We asked whether and how RT heterogeneity fluctuates throughout S phase. We generated a sigmoid model^29^ and computed the relationship between RT values and *T*_width_ (Extended Data Fig. 6a), which quantifies the time difference at which 25–75% of cells replicated a given genomic bin^10,30^, for each stage. The *T*_width_ value thus reflects the variation in RT across cells within the same stage. *T*_width_ values decreased during development, indicating an overall more coordinated RT programme (Fig. 3a). However, *T*_width_ increased again for ICM, reflecting the heterogenous nature of the ICM preceding its segregation into epiblast and primitive endoderm lineages (Fig. 3a). Regions replicating early and late were relatively homogenous (Extended Data Fig. 6b). Overlapping of RT features onto *T*_width_ values indicated that RT peaks and RT troughs are less heterogeneous compared with TTRs (Fig. 3b). In addition, RT peaks and RT troughs are remarkably uniform across cells of the same stage. We also calculated *M*, which is the replication score at which 50% of cells have replicated a given genomic bin. Thus, the distribution of *M*-values indicates how well partitioned into early and late are RT values across the genome. *M* values for mouse ES cells depicted a clear bimodal distribution, reflecting well-defined early and late RT patterns (Fig. 3c). This was not the case for early embryonic stages (Fig. 3c). Instead, a bimodal distribution became apparent after the 2-cell stage, reflecting the emergence of a RT programme that separates the genome towards early (earlier) and late (later) RT values (Fig. 3c and Extended Data Fig. 6c). We conclude that RT heterogeneity fluctuates during S phase within each developmental stage in the same manner as it does in all previously studied systems, and that segregation between early and late RT values increases as development proceeds.

## Consolidation of RT is characterized by specific changes in histone modifications

**Fig. 4:**
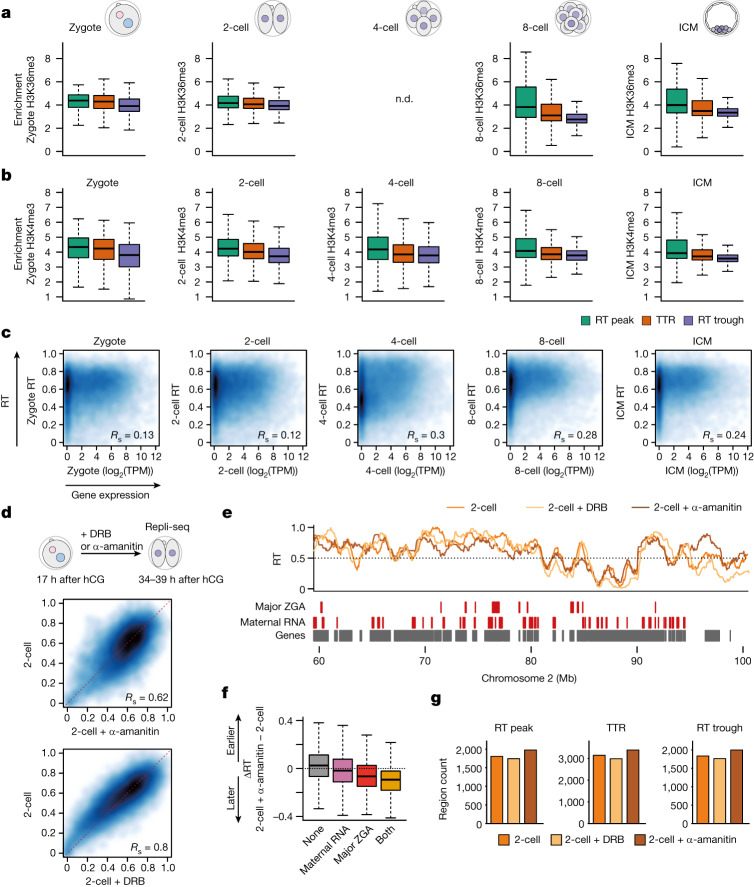
Consolidation of RT is characterized by specific changes in histone modifications at RT troughs and RT peaks and is influenced by ZGA. **a**, H3K36me3 coverage at the indicated replication features at different embryonic stages. **b**, H3K4me3 coverage at replication zones at each embryonic stage. **c**, Smoothed scatterplots showing correlation between RT values and transcript abundance (log_2_(TPM)) at the indicated embryonic stages. Spearman’s correlation is indicated. Note that Spearman’s *R* measures not only linear, but also monotonic, relationships and is robust to outliers. **d**, Smoothed scatterplots showing correlation of RT values between control 2-cell embryos and those treated with α-amanitin (top) or DRB (bottom). DRB or α-amanitin was applied continuously from the zygote stage (17 h after human chorionic gonadotropin (hCG)) until collection at the 2-cell stage, to block both minor and major ZGA, as indicated in the schematic. Spearman’s correlation is indicated. **e**, RT profiles of 2-cell embryos overlayed with those from α-amanitin- and DRB-treated 2-cell embryos. Genomic positions of indicated gene classes according to DBTMEE^54^ are shown as rectangles. **f**, Box plots showing the difference in RT values (ΔRT) between α-amanitin-treated and untreated 2-cell embryos at genomic bins overlapping only major ZGA genes, only maternal RNA genes or both gene classes compared with non-overlapping bins. **g**, Number of replication features in control 2-cell embryos and in embryos treated with α-amanitin or DRB. Box plots show median and IQR, whiskers depict the lowest and highest values within 1.5× IQR. n.d., not determined (data not available).

**Extended Data Fig. 7:**
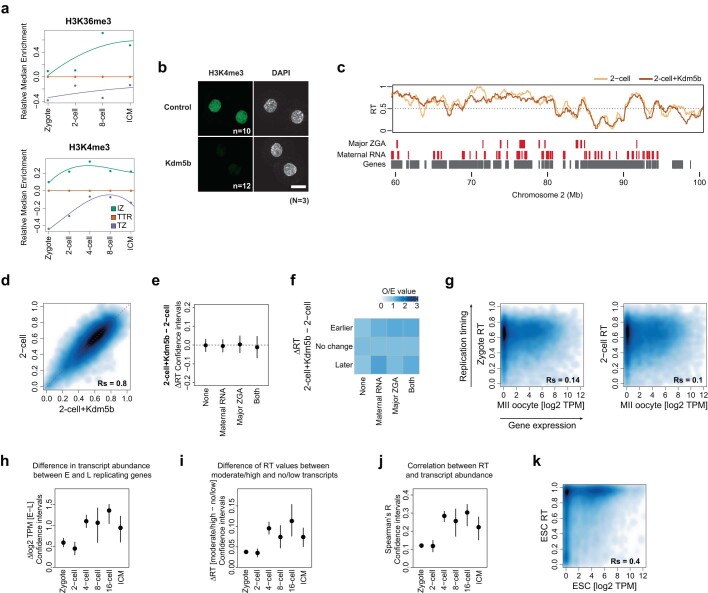
Correlation between RT values in zygotes and maternal transcripts and analysis of histone modifications in RT peaks, TTRs and RT troughs. **a**. Kinetics of the relative changes in the enrichment of H3K36me3 and H3K4me3 at RT peaks and RT troughs normalised to TTRs from the zygote to the morula stage. **b**. Immunostaining of histone H3 lysine 4 trimethylation (H3K4me3) after overexpression of Kdm5b. Representative maximal projection images are shown. Total number of embryos (n) analysed in each condition from three independent experiments (N) are shown. Scale bar, 25 μm. **c**. RT profiles of 2-cell stage embryos overlayed with those from Kdm5b-overexpressed 2-cell embryos. Genomic positions of indicated gene classes according to DBTMEE^54^ are shown as rectangles. **d**. Smoothed scatterplot of RT values in normal 2-cell embryos versus Kdm5b-overexpressed 2-cell embryo. Spearman’s correlation (Rs) is indicated. **e**. Confidence intervals for the changes of RT (ΔRT) between Kdm5b-overexpressed and untreated 2-cell embryos of genomic bins containing maternally expressed genes or Major ZGA genes. ‘Both’ refers to bins containing ZGA genes and maternally expressed genes, whereas ‘None’ does not overlap with any of the two categories. **f**. Enrichment of genomic regions displaying a significant change in RT upon Kdm5b expression in bins containing maternally expressed genes or Major ZGA genes. ‘Both’ refers to bins containing ZGA genes and maternally expressed genes, whereas ‘None’ does not overlap with any of the two categories. Observed over expected number of bins is shown (O/E). **g**. Smoothed scatterplots showing correlations between transcript levels (log2 TPM) of Metaphase II (MII) stage oocytes with the RT values of zygote and 2-cell stage embryos. Rs indicates Spearman’s R. **h**. Confidence intervals for the difference of transcript levels (Δlog2 TPM) between early (E) vs. late (L) replicating genes. Genomic bins with an RT value greater than 0.5 were considered as Early and with RT value lower than 0.5 as Late. **i**. Confidence intervals for the difference of replication timing (ΔRT) between genes with moderate/high vs. no/low transcript levels. Genes with a transcript level (log2 TPM) greater than 1 were considered moderate/high and with a value lower than 1 as no/low expressed. **j**. Confidence intervals for the Spearman’s correlation between RT and transcript abundance. **k**. Smoothed scatterplot showing correlation between transcript levels (log2 TPM) and RT values in mouse ES cells. Rs indicates Spearman’s R. In e, h-j the dot represents the mean of 1000 bootstrapped values. Error bars indicate the 95% bootstrap confidence interval.

The relationship between RT and transcription remains unclear, with often contradictory reports on RT instructing transcription or vice versa^9,31^. Because the embryo starts transcription de novo following a period of transcriptional silence in the germline, the embryo provides an outstanding opportunity to disentangle the role of transcriptional activation in the establishment of RT. Our above results indicate that the RT programme becomes progressively more defined, particularly after the 2-cell stage (Fig. 1d,h), which corresponds to the time of ZGA^17^. Thus we first asked whether chromatin features of active transcription relate to the progressive definition of RT. H3K36me3 became enriched at RT peaks from the 8-cell stage (Fig. 4a) (no available data for H3K36me3 at the 4-cell stage), indicating that H3K36me3 marks emerging RT peaks (Extended Data Fig. 7a). Whereas H3K36me3 is associated with gene bodies and is thus typically excluded from replication origins in other cells^23^, H3K36me3 does not necessarily reflect transcription elongation kinetics during development^32^ and thus our findings may reflect specific embryonic chromatin features. H3K4me3 levels were relatively stable across RT peaks, TTRs and RT troughs, with slightly higher levels at RT peaks and a depletion in RT troughs in zygotes and 2-cell embryos compared with later stages (Fig. 4b and Extended Data Fig. 7a). Because oocytes have distinctive broad H3K4me3 domains, which are remodelled by demethylases KDM5A/5B upon ZGA^33,34^, we asked whether H3K4me3 inheritance is linked to RT in embryos. For this we expressed KDM5B^14^, known to remove H3K4me3 broad domains^34^, in mouse zygotes and performed scRepli-seq at the 2-cell stage (Extended Data Fig. 7b). RT profiles following KDM5B expression showed a similar global pattern in control of 2-cell embryos (Extended Data Fig. 7c,d). In addition, KDM5B expression did not affect RT of major ZGA genes, nor of genes expressed in oocytes (Extended Data Fig. 7e,f), indicating that removal of H3K4me3 following fertilization does not majorly impact RT at regions containing major ZGA genes.

Next we examined whether RT relates to gene expression levels. Genome-wide correlation of RT values and steady-state transcript abundance were low in zygotes and 2-cell embryos (Spearman’s correlation, *R*_s_; Fig. 4c). In fact, RT in zygotes and 2-cell embryos correlated similarly with the transcriptome of non-fertilized oocytes and zygotes (Extended Data Fig. 7g). This suggests that either the presence of maternally inherited transcripts from oocytes, which dominates the early transcriptome, overrides a possible relationship with RT or that transcriptional activity does not correlate strongly with RT at these stages. We favour the latter interpretation because 2-cell embryos, which undergo massive transcriptional activation and degradation of maternal transcripts, show a similar correlation between their RT and transcriptome to zygotes (Fig. 4c). Both transcript abundance and RT values change significantly during developmental progression and thus the increasing correlation between RT and transcription during development stems from changes in both transcript abundance and RT (Extended Data Fig. 7h,i). From the 4-cell stage, the correlation between RT and transcript levels increases and the typical relationship between transcription and early replication emerges, with genes expressed at high levels replicating early (Fig. 4c and Extended Data Fig. 7i). Indeed, the correlation between transcript abundance and RT values is significantly greater from the 4-cell stage onwards (Extended Data Fig. 7j). This correlation is similar to ES cells, albeit at a lower extent (Extended Data Fig. 7k). These data show that the known correlation between RT and gene expression emerges gradually from the 4-cell stage, with genes showing the highest expression replicating early during S phase.

## RNA polymerase II at ZGA contributes to fine-tuning of the RT programme

**Extended Data Fig. 8:**
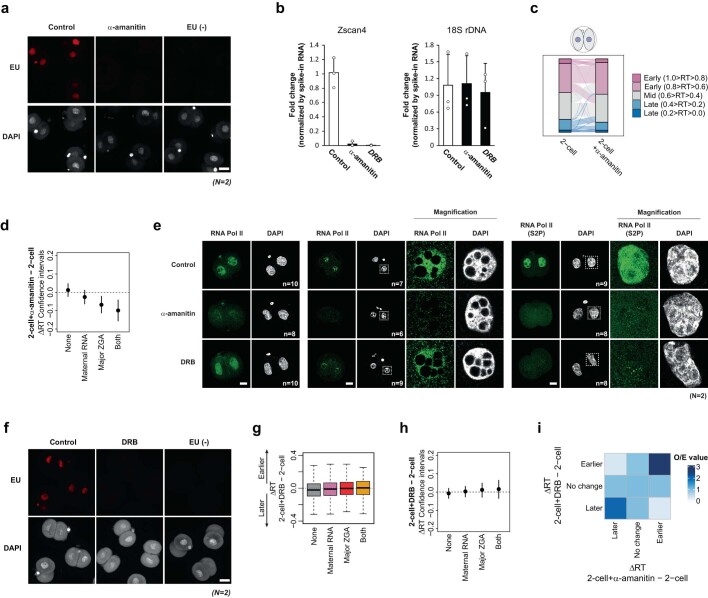
Effect of RNA Pol II inhibition by α-amanitin and DRB on the embryonic RT programme. **a**. Visualisation of global transcription during minor and major ZGA by EU click chemistry and efficient inhibition of ZGA using α-amanitin. Representative embryos of a total of 24 (control), 19 (α-amanitin treated) or 19 non-EU treated embryos (EU-) are shown. Scale bar, 50 μm. **b**. Taqman RT-qPCR analysis for Zscan4 cluster and rDNA after α-amanitin and DRB treatment. Barplots show mean ± s.d and dots indicate the values of independent biological replicates. **c**. Alluvial plot indicating the RT values categorised in 5 groups from the earliest (1.0 ≥ RT > 0.8) to latest RT (0.2 > RT ≥ 0.0) across the genome in control 2-cell embryos and their changes upon α-amanitin treatment. **d**. Confidence intervals for the changes in RT (ΔRT) upon α-amanitin treatment of genomic bins containing maternally expressed genes or major ZGA genes. ‘Both’ refers to bins containing ZGA genes and maternally expressed genes, whereas ‘None’ does not overlap with any of the two categories. **e**. Immunostaining of RNA Pol II using an antibody recognizing all forms of RNA Pol II or an antibody against its CTD Serine 2 phosphorylated form (S2P) after α-amanitin or DRB treatment with (right) and without (left) Triton pre-extraction. Representative single confocal sections are shown. Total number of embryos (n) analysed in each conditions from two independent experiments (N) are shown. Scale bars, 25 μm. We note that α-amanitin leads to degradation of RNA PolII in our experimental conditions. **f**. Visualisation of global transcription during minor and major ZGA by EU click chemistry and efficient inhibition of ZGA upon DRB treatment. Representative embryos from two independent experimetns (N) are shown. Scale bar, 50 μm. **g**. Difference of RT values (ΔRT) between DRB-treated and untreated 2-cell embryos at genomic bins overlapping only major ZGA genes, only maternal RNA genes, or both genes compared to non-overlapping bins (None). Box plots show median and the interquartile range (IQR), whiskers depict the smallest and largest values within 1.5 ×IQR. **h**. Confidence intervals for the changes in RT (ΔRT) upon DRB treatment of genomic bins containing maternally expressed genes or Major ZGA genes. ‘Both’ refers to bins containing ZGA genes and maternally expressed genes, ‘None’ does not overlap with any of the two categories. **i**. Enrichment of genomic regions displaying significant changes in RT upon α-amanitin treatment with bins that display changes in RT upon DRB treatment in 2-cell stage embryos. Observed over expected number of bins is shown (O/E). In d and h, the dot represents the mean of 1000 bootstrapped values. Error bars indicate the 95% bootstrap confidence interval.

**Extended Data Fig. 9:**
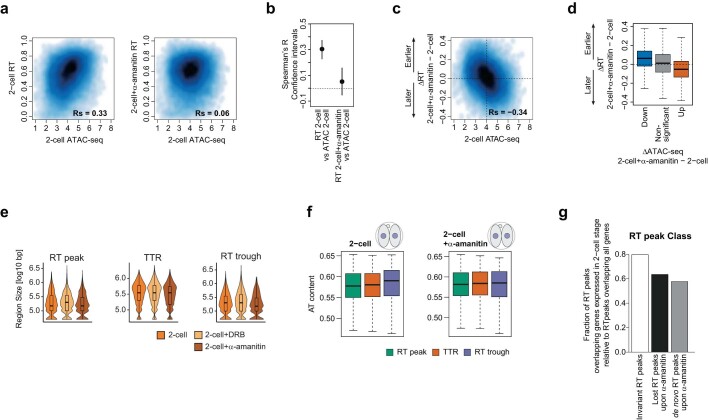
Relationship between ATAC-seq and RT changes upon transcriptional inhibition. **a**. Smoothed scatterplot showing correlation between ATAC-seq signal and RT values in 2-cell stage embryos (left) and in α-amanitin treated 2-cell stage embryos (right). Rs indicates Spearman’s R. **b**. Pairwise Spearman´s correlation coefficients (R) between RT and ATAC-seq signal in untreated and in α-amanitin treated 2-cell stage embryos. Error bars indicate the 95% bootstrap confidence interval. Dot represents the mean of 1000 bootstrapped values. **c**. Smoothed scatterplot depicting the difference of RT values (ΔRT) between α-amanitin treated and untreated 2-cell embryos against ATAC-seq signal in control 2-cell stage embryos. **d**. Difference of RT values (ΔRT) at genomic bins that significantly lose accessibility (down), gain accessibility (up) or remain unchanged (non-significant) upon α-amanitin treatment in 2-cell stage embryos. Box plots show median and the interquartile range (IQR), whiskers depict the smallest and largest values within 1.5 ×IQR. **e**. Size of RT peaks, TTRs and RT troughs in control versus α-amanitin or DRB treated 2-cell embryos. **f**. A + T content in RT peaks, TTRs, and RT troughs in 2-cell and α-amanitin treated 2-cell embryos. Box plots show median and the interquartile range (IQR), whiskers depict the smallest and largest values within 1.5 ×IQR. **g**. Fraction of RT peaks containing genes expressed at the 2-cell stage relative to all genes in RT peaks specific to 2-cell stage embryos upon α-amanitin treatment (de novo), in RT peaks specific to control 2-cell stage embryos (lost) and RT peaks present in both 2-cell control and α -amanitin treated embryos.

We next addressed directly whether transcription regulates the establishment of RT. We incubated zygotes with α-amanitin under conditions that prevent minor and major ZGA but do not affect RNA polymerase (Pol) I transcription, and performed scRepli-seq at the 2-cell stage (Extended Data Fig. 8a,b). Evaluation of RT at later stages is not feasible because inhibition of ZGA prevents development beyond the 2-cell stage^17^. RT values in α-amanitin-treated embryos showed a moderate correlation with control embryos (Fig. 4d), suggesting that prevention of ZGA with α-amanitin may affect RT at the 2-cell stage. Indeed, we observed changes in RT towards earlier and later following α-amanitin treatment (Extended Data Fig. 8c). Further examination showed localized RT changes in α-amanitin-treated embryos (Fig. 4e), with a statistically significant delay in RT of genomic bins overlapping with major ZGA genes but not of regions containing genes expressed in oocytes (maternal genes) or control regions (Fig. 4f and Extended Data Fig. 8d). To better understand how transcription at ZGA affects RT, we sought to distinguish the effects of general transcription inhibition versus transcription elongation. We took advantage of another RNA Pol II inhibitor, 5,6-dichlorobenzimidazone-1-β-d-ribofuranoside (DRB), which inhibits transcriptional elongation by inhibition of RNA Pol II Ser2 phosphorylation, whereas α-amanitin results in full transcriptional inhibition^35^, including via RNA Pol II degradation (Extended Data Fig. 8e,f). DRB treatment during the same period as α-amanitin led to milder changes in RT compared with α-amanitin (Fig. 4d,e). Interestingly, DRB did not significantly change RT of genomic bins containing ZGA genes (Extended Data Fig. 8g,h), suggesting that transcriptional elongation of ZGA genes does not affect their RT. However, DRB and α-amanitin led to similar changes in RT of regions without genes expressed at the 2-cell stage (Fig. 4e and Extended Data Fig. 8i). Thus, we next explored whether other chromatin features relate to the RT phenotype following ZGA inhibition. Prevention of ZGA with α-amanitin alters accessibility in 2-cell embryos^36,37^. Analysis of assay for transposase-accessible chromatin using sequencing (ATAC-seq) datasets showed a significant, positive correlation with RT in 2-cell embryos, indicating that regions replicating early are, in general, more accessible than those that replicate late (Extended Data Fig. 9a,b). This correlation was lost following α-amanitin treatment (Extended Data Fig. 9a,b). Globally, the changes in RT elicited by α-amanitin anticorrelated with sites of genome-wide accessibility in 2-cell control embryos (Extended Data Fig. 9c). Indeed, we find that regions that gain ATAC-seq signal following α-amanitin treatment become replicated later; likewise, regions that lose accessibility become replicated earlier (Extended Data Fig. 9d).

To further understand how transcription during ZGA influences RT, we examined RT features in 2-cell embryos treated with α-amanitin or DRB. Prevention of transcription at ZGA using α-amanitin, but not DRB, led to more TTRs, RT peaks and RT troughs with a concomitant decrease in the size of RT troughs (Fig. 4g and Extended Data Fig. 9e). The increase in their number and the smaller RT troughs suggests a more fragmented, less consolidated RT programme after α-amanitin treatment. These data also suggest that replication may initiate and terminate at different locations in the absence of embryonic transcription. In support of this, RT troughs in α-amanitin-treated embryos do not show AT content enrichment, in contrast to controls (Extended Data Fig. 9f). In addition, de novo RT peaks in α-amanitin-treated embryos contain fewer genes normally expressed at the 2-cell stage compared with those insensitive to α-amanitin (Extended Data Fig. 9g). Thus, perturbation of RNA Pol II globally at ZGA contributes to fine-tuning of initiation and termination sites at the 2-cell stage.

**Extended Data Fig. 10:**
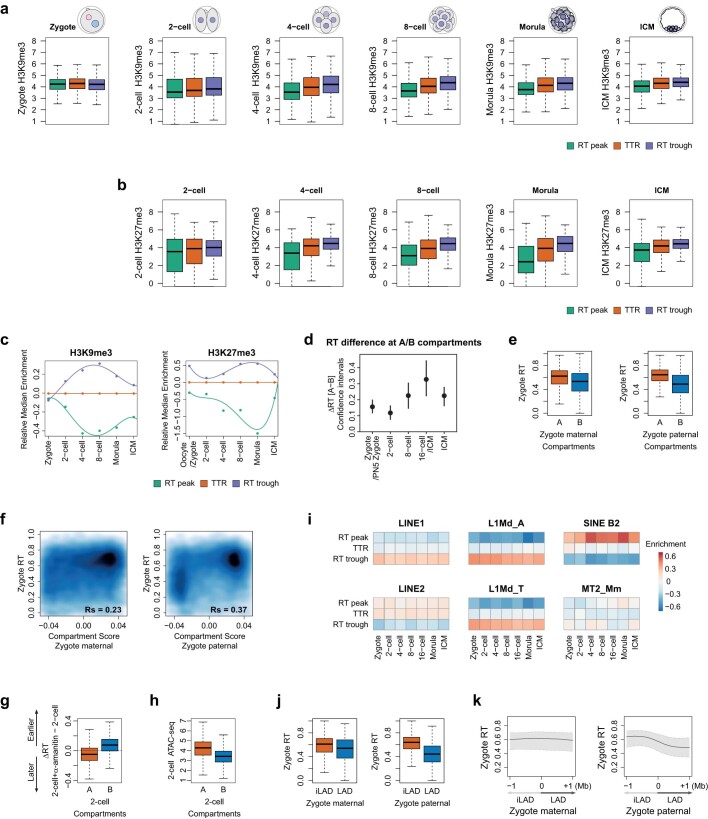
Characterisation of silent chromatin features of the embryonic replication programme and of the parental RT differences of LADs and compartments. **a**., **b**. Box plots depicting H3K9me3 (a) or H3K27me3 (b) coverage at the indicated replication features at different embryonic stages. **c**. Kinetics of the relative changes in the enrichment of histone modifications at RT peaks and RT troughs normalised to TTRs from the zygote to the blastocyst stage ICM. The ‘oocyte/zygote’ time point indicates H3K27me3 data from oocytes, before fertilisation, and RT from zygotes (after fertilisation). **d**. Analysis to determine statistical significance on the RT differences between A and B compartments based on confidence intervals. Confidence intervals for the difference of replication timing (ΔRT) between A and B compartments. Error bars indicate the 95% bootstrap confidence interval. Dot represents the mean of 1000 bootstrapped values. **e**. Box plots of zygote RT values in maternal (left) and paternal (right) A and B compartments. **f**. Smoothed scatterplots showing the correlation between zygote RT values and maternal and paternal compartment scores. **g**. Box plot depicting the difference of RT values (ΔRT) between α-amanitin treated and untreated 2-cell embryos in A- and B-compartments. **h**. Box plot depicting the ATAC-seq signal in A- and B-compartments in untreated 2-cell stage embryos. **i**. Enrichment of the main families of transposable elements across replication features during early development. Color key indicates the number of overlapping TEs relative to randomly shuffled. **j**. Box plots showing RT values of zygotes within the corresponding zygotic maternal (left) and paternal (right) iLADs and LADs. **k**. Composite plot showing RT values of zygotes plotted against maternal and paternal zygotic LADs. The zero indicates the position of the LAD/iLAD boundaries. Shading shows IQR and the line indicates the median. In a, b, e, g, h, j the box plots show median and the interquartile range (IQR), whiskers depict the smallest and largest values within 1.5 ×IQR.

Finally, we characterized silent chromatin features of the embryonic replication programme. RT troughs contain higher levels of H3K9me3 compared with RT peaks and, to a lesser extent, with TTRs, but these differences emerge only from the 2-cell stage and H3K9me3 levels across RT peaks, TTRs and RT troughs are equivalent in zygotes (Extended Data Fig. 10a). H3K27me3 levels are lowest at RT peaks at all developmental stages and, similarly to H3K9me3, RT peaks and RT troughs acquire gradually different histone modifications during development, with RT peaks showing a depletion of H3K27me3 compared with TTRs and RT troughs by the morula stage (Extended Data Fig. 10b,c). These findings may relate to the progressive heterochromatin maturation of early embryos^38,39^. Overall, maturation of the RT programme is accompanied by a progressive, relative increase in H3K9me3 at RT troughs and a gradual decrease at RT peaks.

## Organization into LADs and inter-LADs precedes partitioning of early and late replication

**Fig. 5:**
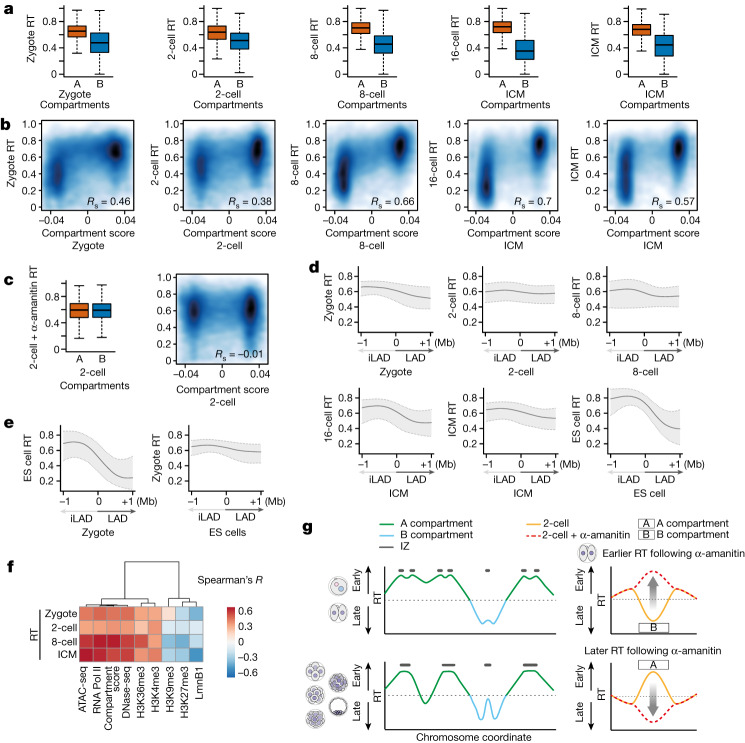
The distinctive RT between A and B compartments is dependent on ZGA, and three-dimensional genome organization precedes partitioning of early- and late-replication dynamics. **a**, Box plots showing RT values in A and B compartments at the indicated stages. Note that, because HiC (high-throughput chromosome conformation capture) data for the 16-cell stage were unavailable, we used the closest developmental stage (ICM) for this comparison. **b**, Smoothed scatterplots showing correlation between RT values and compartment score at the indicated stages. Spearman’s correlation is indicated. **c**, Box plots showing RT values in A and B compartments (left) and correlation between RT values and compartment score (right) in α-amanitin-treated, 2-cell-stage embryos. **d**, Composite plots depicting RT values computed against LADs and iLADs at their corresponding developmental stage. Zero indicates the position of LAD–iLAD boundaries. Because DamID data for the 16-cell stage were not available, we used the closest developmental stage (ICM) for this comparison. **e**, Composite plots depicting RT values of mouse ES cells plotted against zygotic LADs (left) and RT values of zygotes against LADs in ES cells (right). Zero indicates the position of LAD–iLAD boundaries. **d**,**e**, Shading and lines indicate IQR and median, respectively. **f**, Correlation (Spearman’s *R*) heatmap between RT and distinctive chromatin features. When data for the same stage as RT are not available, those of the closest stage are used for analysis. **g**, Model summarizing our findings indicating progressive resolution of RT following the 2-cell stage. Left, RT peaks merge over time, resulting in changes in both number and size. Right, the effect of ZGA inhibition on RT and its relationship to A and B compartments. **a**,**c**, Box plots show median and IQR, whiskers depict the lowest and highest values within 1.5× IQR.

Finally we investigated the dependency between three-dimensional genome architecture and the establishment of RT. In differentiated and stem cells, early and late replication correlate with the A and B compartments, respectively^3,40^, and TADs tend to correspond to replication domains^2^. However, because TADs are not clearly detected in early cleavage stages^14,16^ we focused on compartments and asked whether the A and B compartments already differ in their RT at the earliest developmental stages. A compartments consistently showed an earlier RT profile compared with B compartments (Fig. 5a and Extended Data Fig. 10d). The distinction between early and late RT values in both compartments was less pronounced in zygotes and became clearer as development proceeds (Fig. 5a). In line with only minor differences in the RT of parental genomes (Fig. 2), RT values were only slightly different in maternal and paternal A and B compartments (Extended Data Fig. 10e). RT differed more between paternal A and B compartments than in maternal compartments, potentially because of the weaker structure of the latter^14–16^ (Extended Data Fig. 10e,f). The difference in RT values between A and B compartments increased during development due to both better segregation of RT values and increase in compartment score (Fig. 5b). Inhibition of ZGA with α-amanitin completely eliminated RT differences between A and B compartments but the compartment score remained similar (Fig. 5c)^14^. Globally, A compartments replicated later and B compartments replicated earlier in α-amanitin-treated embryos compared with controls (Extended Data Fig. 10g). Because B compartments are less accessible than A compartments (Extended Data Fig. 10h), these observations can be explained by our results indicating that α-amanitin leads to a shift towards earlier replication of less accessible regions. We conclude that partitioning of early and late RT during early development coincides with the maturation of A and B compartments. In addition, whereas ZGA does not contribute to compartment strength^14^, transcriptional inhibition equalizes differences in RT between compartments.

The genetic constitution of mammalian A and B compartments is largely demarcated by repetitive elements^41,42^, which are expressed in the mouse embryo^43,44^. Namely, LINE1 are highly transcribed at the 2-cell stage^43,45^ and are enriched in LADs and B compartments^41,42,46^. In fact, LINE1 and SINE segregate mostly exclusively into B and A compartments, respectively^41^. Thus we investigated the replication features of major transposable element families. Overall, LINE1 were enriched in RT troughs and depleted in RT peaks (Extended Data Fig. 10i). This enrichment was stronger for evolutionarily young LINE1, L1Md_A and L1Md_T, contrasting with older LINE2, which showed depletion from RT troughs (Extended Data Fig. 10i). SINE B2 are enriched in RT peaks and depleted in RT troughs, and this tendency became clearer from the 4-cell stage (Extended Data Fig. 10i). MERV-L (MT2_Mm), highly transcribed in 2-cell embryos^44,47^, was more homogeneously distributed across RT peaks, TTRs and RT troughs. However, MERV-L enrichment in RT features, albeit low, changed throughout development (Extended Data Fig. 10i). Thus the RT of domains containing MERV-L, unlike LINEs, is dynamic (Extended Data Fig. 10i). Indeed, a change in RT of MERV-L occurs during reprogramming of 2-cell-like cells (2CLCs)^48^.

Finally we examined the relationship between LADs and RT. LADs are established in zygotes immediately following fertilization and are reorganized during preimplantation development, but a large proportion of LADs remains constant and is similar to ES cell LADs^13^. In general, LADs, unlike inter-LADs (iLADs), replicate late^2,49^. However, and in sharp contrast to ES cells, RT in zygotes is not clearly distinguishable between LADs and iLADs (Fig. 5d). Zygotic LADs differ between parental genomes^13^ and, accordingly, paternal LADs and iLADs exhibit a slight segregation of RT values and maternal ones to a lesser extent (Extended Data Fig. 10j). RT in zygotes did not exhibit a strong bias towards either paternal or maternal LADs/iLADs (Extended Data Fig. 10k). The separation of RT values in LADs and iLADs increases as development proceeds, reaching a clear distinction in ES cells (Fig. 5d). These observations raise the possibility that nuclear organization into LADs and iLADs temporally precedes establishment of the RT programme. To address this, we asked whether RT in ES cells corresponds to LADs/iLADs in zygotes. Remarkably, RT values in embryonic stem cells plotted against the LAD boundaries of zygotes indicated a clear demarcation of RT in embryonic stem cells according to zygotic LAD boundaries (Fig. 5e), indicating that LAD organization in zygotes predisposes RT at later stages of development. In contrast, plotting the RT values of zygotes over ES cell LAD boundaries did not show such a correlation (Fig. 5e). We conclude that organization of LADs and iLADs at the beginning of development precedes the partitioning of early- and late-replication dynamics.

## Discussion

Our data indicate that the establishment of RT occurs progressively following fertilization, hand-in-hand with the gradual acquisition of distinctive chromatin features and similarly to other epigenomic features (Fig. 5f). The less well-defined, more heterogeneous RT programme in zygotes and 2-cell embryos may reflect a higher plasticity in the chromatin structure in general and could also be related to changes in histone deposition occurring at these stages^50^. RNA Pol II in zygotes and 2-cell-stage embryos contributes to the definition of RT. The comparatively milder effects on RT elicited by DRB compared with α-amanitin suggest that RNA Pol II itself influences the RT programme in 2-cell-stage embryos to a greater extent than transcriptional elongation. Although further investigation is warranted to determine whether additional, non-transcription-related effects contribute to these observations—for example via structural proteins^51^—our findings align with work showing that ZGA transcription may be less affected by DRB than by α-amanitin^34,52^.

The correlation between transcriptional activity and RT emerges after the 2-cell stage, coinciding with progressive lengthening of the G1 phase^53^, known to be important in the definition of RT^6^. Although we observed large-scale changes in RT, for example, with around 20% of the genome switching from early to late RT during preimplantation development, fine-scale changes through the gradual acquisition of histone modifications are also likely to contribute to tuning of RT as cell types emerge. Remarkably, our data indicate that transcription and RNA Pol II function contribute to the definition of the epigenetic features of compartments, in this case their RT (Fig. 5g), but not to their segregation^14^. Our observations that the genome structuring into LADs and iLADs precedes the partitioning of RT at later developmental stages establishes an exciting temporal dependency between these two pillars of the epigenome.

Our work lays the foundations for understanding how genome replication is regulated during development and sheds light on how the epigenome is remodelled at the beginning of mammalian development.

## Methods

### Embryo collection and culture

All experiments were performed under the authorization of the authorities from Upper Bavaria (Tierversuchsantrag von Regierung von Oberbayern). The temperature, humidity and light cycle of mouse cages were maintained at 20–24 °C, 45–65% and 12/12 h dark/light, respectively. F_1_ female mice (C57BL/6J × CBA) under 10 weeks of age were superovulated by intraperitoneal injection of 10 U of pregnant mare serum gonadotropin, followed by 10 U of hCG 48 h later, and were then mated with DBA/2J male mice. Zygotes were collected from the oviduct and cumulus cells removed following brief incubation in M2 medium containing hyaluronidase (Sigma-Aldrich). Zygotes were placed in drops of KSOM (potassium simplex optimized medium) and cultured at 37 °C with 5% CO_2_ as previously described. For induction of parthenogenetic embryos, MII-stage oocytes were collected, as described above, from superovulated females without mating. Following removal of cumulus cells, oocytes were treated with 10 mM Sr^2+^ for 2 h in Ca^2+^-free CZB medium and then incubated in KSOM. For generation of IVF-derived zygotes, MII oocytes from F_1_ female mice (C57BL/6J × CBA) were inseminated with activated spermatozoa obtained from the caudal epididymides of adult DBA/2 J male mice.

### Detection of 5-ethynyl-2′-deoxyuridine incorporation

Cells were incubated with 50 μM 5-ethynyl-2′-deoxyuridine (EdU) for 1 h for each time window, as indicated, and processed for quantification of signal intensity. Incorporated EdU was visualized by Click-iT chemistry (Thermo Fisher Scientific) followed by permeabilization as described in the manufacturer’s instructions. Images were acquired on a SP8 confocal laser-scanning microscope (Leica). EdU was coupled to Alexa 594 and images acquired with a Plan-Apochromat ×63/1.4 numerical aperture 1.4 oil-immersion objective (Leica) at 561 nm excitation.

### Analysis of EdU incorporation

To quantify EdU incorporation we manually cropped confocal stacks containing several embryos so that each image contained only one single embryo. Only embryos that looked fertilized and with normal pronuclei following visual inspection were included in this analysis. From embryo images we then automatically obtained the maximum intensity value in the EdU channel of the whole stack by ImageJ (v.1.53k) with a custom-made ImageJ macro. We plotted and analysed the resulting EdU intensity values for each time bin with R.

### Inhibition of ZGA

For inhibition of both minor and major ZGA, embryos were treated with either 0.1 mg ml^−1^ α-amanitin or 100 μM DRB from the zygote stage at 17 h after hCG injection until their collection for single-cell Repli-seq at the 2-cell stage. Validation of the α-amanitin effect on transcriptional silencing was done using a Click-iT RNA Alexa Fluor 594 Imaging Kit (Thermo Fisher Scientific) at the 2-cell stage (at 40 h after hCG injection).

### Gene expression analyses following treatment with α-amanitin and DRB

Twelve embryos were treated with either 0.1 mg ml^−1^ α-amanitin or 100 μM DRB from 17 to 40 h after hCG to inhibit both minor and major ZGA, then flash-frozen in liquid nitrogen in 5 μl of 2× reaction buffer (CellsDirect One-Step qRT–PCR kit, no. 11753100, Thermo Fisher). Next, 0.5 μl of a 1:200 dilution of ERCC spike-in mix (Thermo Fisher) was added to each group and TaqMan Gene Expression assays were performed according to previous work^38^. Complementary DNA was diluted tenfold before analysis with Universal PCR Master Mix and TaqMan Gene Expression assays (Applied Biosystems). All raw *C*_t_ values were normalized by those acquired from the ERCC spike-in specific primer set, and relative expression levels of each gene were determined by the ddCt method. We assigned *C*_t_ values below the detection range as expression level 0. Primers and probes for ribosomal DNA (*Hsa1*) were produced by TIB MolBiol (custom design)^45^. Primers and probes for Zscan4 cluster and ERCC spike-in were purchased from Applied Biosystems.

### Immunostaining following either treatment by α-amanitin and DRB or expression of KDM5B

Embryos were treated with either 0.1 mg ml^−1^ α-amanitin^55,56^ or 100 μM DRB from 17 to 40 h after hCG and fixed with 4% paraformaldehyde (PFA) for 20 min at room temperature. For KDM5B expression, 2 μg μl^−1^ KDM5B of in vitro synthesized messenger RNA was microinjected into zygotes at 18 h after hCG and fixed with 4% PFA for 20 min at room temperature at 48 h after hCG, similar to previous experiments^13,33^. Embryos were then permeabilized with 0.5% Triton X-100 containing PBS for 20 min. For immunostaining following Triton pre-extraction, embryos were first permeabilized with pre-extraction buffer (50 mM NaCl, 3 mM MgCl_2_, 300 mM sucrose, 25 mM HEPES, pH adjusted to 7.4) with 0.5% Triton X-100 for 10 min on ice and washed three times in pre-extraction buffer before fixing in 4% PFA at room temperature for 20 min. Following blocking for 1 h at room temperature in blocking solution (5% normal goat serum in PBS), embryos were incubated with either anti-RNA polymerase II (no. sc-899, 1:100), anti-RNA polymerase II CTD repeat YSPTSPS (phospho S2, no. ab5095, 1:1,000) or anti-H3K4me3 (Diagenode, no. C15410003, 1:250) antibody in blocking solution overnight at 4 °C. Embryos were incubated for 1.5 h at room temperature in blocking solution containing goat anti-rabbit IgG highly cross-adsorbed secondary antibody, Alexa Fluor 488 (Thermo Fisher Scientific, no. A11034, 1:1,000). After washing, embryos were mounted in Vectashield (Vector Laboratories). Confocal microscopy was performed using a ×40 oil objective on an SP8 confocal microscope (Leica) and images acquired with LAS X software.

### Repli-seq

Single-cell Repli-seq was performed as previously described^19^ based on ref. ^5^. In brief, early-stage zygotes were collected and cultured until they reached the S phase at each developmental stage, based on their time following hCG injection. Embryos were collected at different time points at each developmental stage to achieve sampling over the entire S phase. Collection times are indicated in Supplementary Table 1. For parthenogenetic embryos and IVF-derived zygotes, the timing of S phase was calculated based on the time elapsed since activation and insemination, respectively. For KDM5B experiments, 2 μg μl^−1^ KDM5B of in vitro synthesized mRNA was microinjected into zygotes at 18 h after hCG as previously described^13^. For each developmental stage, embryos were obtained from several litters and embryos from different litters were collected across different dates to ensure robust data collection. The number of mice used for collection of samples for each developmental stage is indicated in parentheses, as follows: zygote (20), 2-cell (30), 4-cell (27), 8-cell (20), 16-cell (15), morula (16), ICM (19), parthenotes (14), IVF zygotes (14), 2-cell + α-amanitin (14), 2-cell + DRB (24) and 2-cell + KDM5B (24). Zona pellucida was removed by exposure to acid Tyrode, and each blastomere was dissociated by gentle pipetting following trypsin treatment. For Repli-seq with physically isolated pronuclei we distinguished maternal and paternal pronuclei based on their size and relative position to the second polar body, and isolated them using micromanipulation. The remaining zygote containing a single pronucleus was also collected following removal of the polar body so that both pronuclei from the same zygote were further processed for Repli-seq. ICM cells were collected following trypsin digestion as previously described^57^, with repeated oral pipetting in 0.5% trypsin and 1 mM EDTA; collection times are indicated in Supplementary Table 1. To distinguish ICM from trophectoderm cells, blastocysts were labelled with Fluoresbrite YG Microspheres (0.2 μm, Polysciences) before incubation with trypsin, and individual cells were sorted according to either positive (trophectoderm) or negative (ICM) fluorescence under a fluorescence microscope following disaggregation. Individual blastomeres or pronuclei were placed in eight-strip PCR tubes containing lysis buffer, and extracted DNA was fragmented by heat incubation. Fragmented DNA was tagged by the universal primer 5′-TGTGTTGGGTGTGTTTGGKKKKKKKKKKNN-3′ and amplified with whole-gene amplification primer sets, which have individual barcodes. This whole-genome amplification procedure was successfully used for single-cell Repli-seq in cell culture^4,5^. Amplified DNA was purified using the QIAquick 96 PCR Purification Kit (QIAGEN), and concentration determined by NanoDrop (Thermo Scientific). Equal amounts of DNA from each sample (up to 96 samples) were pooled and 1 μg of each was ligated with Illumina adaptors using the NEBNext Ultra II DNA Library Prep Kit (NEB). Illumina sequences (NEBNext Multiplex Oligos for Illumina, NEB) were added to adaptor-ligated samples by PCR. Clean-up and size selection of the PCR product was done using SPRIselect (Beckman Coulter), and the quality of the library was confirmed using a 2100 Bioanalyzer with the High Sensitivity DNA Kit (Agilent).

### Single-cell Repli-seq read alignment and quality control filtering

An overview of sample collection, mapping statistics and quality control is included in Supplementary Table 1. The quality control parameters we used were (1) the number of reads, which we set as 750,000 aligned reads as minimum; and (2) a coefficient of variation, which we established as a measure of equal/balanced coverage between chromosomes, thus filtering out potential cells with aneuploidy. At early stages, the reason for failure was equally the low number of reads or a high coefficient of variation (typically due to either lack of reads on a complete chromosome or in fragments of the genome; for example, zygotes 13 and 8 were excluded due to low number of reads and zygote 56 to a high coefficient of variation). At later stages, chromosome imbalances were the most common reason for failure (59 cells with high coefficient of variation versus three with low reads in the blastocyst stage), which reflects the known aneuploidy of cells at this embryonic stage. Sequencing reads were aligned to the mm10 genome using bowtie2 (v.2.3.5)^58^ with the ‘--local’ option. Duplicates were marked using SAMtools (v.1.9) ‘markdup’ as described by SAMtools^59^ documentation (the commands ‘fixmate’ and ‘sort samtools’ were used for this purpose accordingly). Using SAMtools view, reads were filtered by retaining only properly paired reads, removing duplicates and selecting those whose mapping quality was higher than or equal to 20. BED files of the read coordinates were generated with the BEDtools^60^ (v.2.29.0) command ‘bamtobed’. Using BEDtools intersect, read counts were obtained for contiguous 50 kb genomic bins. For each cell the average of the bin counts was calculated for chromosomes 1–19; these 19 values were then next used to calculate the coefficient of variation as standard deviation divided by the mean. Cells with a coefficient of variation greater than 0.1 were removed from analyses due to chromosome imbalance. To maximize the number of samples used, the coefficient of variation was recalculated, excluding chromosomes one at a time. Cells were considered for further analysis if they passed the threshold when only one specific chromosome was removed. This chromosome was subsequently masked in downstream analyses; this filter removes abnormal genotypes and cells with aneuploidy.

### Assignment of replication status

Using the read counts obtained for contiguous 50 kb genomic bins, we used the single-cell Repli-seq bioinformatic pipeline previously described^5^, which we followed with some modifications for each embryonic stage as summarized below. Window counts were first normalized to reads per million, and then each bin by its respective average of all samples within the same stage, aiming to correct for mappability biases intrinsic to genomic regions. Outlier regions were then masked, specifically the windows of the lower fifth percentile and upper first percentile values. To correct for low mappability, windows were segmented with the R package copy number (v.1.28.0, R v.4.0.0)^61^ to retain segments with the highest 95% of values. We did not perform the G1/G2 normalization described previously^5^, but we verified that this did not impact the results of these analyses. In brief, we used the validated mouse ES cell scRepli-seq datasets in ref. ^5^ and ran the analysis pipeline as described in their methods section with and without G1 control cells. Subsequently we compared the generated matrix of ones and zeros (that is, bins replicated and not replicated, respectively) by determining the percentage of windows that remained the same (for example, their 1 or 0 replication state did not change) after running the pipeline versus without G1 control. These analyses showed a high concordance between the two pipelines, with over 91% identity of genomic bins with zeros and ones on average across cells (Extended Data Fig. 1b). Importantly, those cells classified as outliers based on our analysis correspond to those that were removed in the original publication^5^ based on their ‘Removing outlier cells’, and were not considered for further analyses. Data were centred by the mean, scaled by the IQR for each cell and smoothed using a median filter with a running width of 15 windows, followed by segmentation with the R package copynumber. Finally, using the function normalmixEM in the R package mixtools (v.1.2.0)^62^, segmented values were used to fit a mixture model with two components to identify replicated and non-replicated window populations. To do this, two normal distribution functions were used to select a cutting threshold that better separated distributions; this value is located where the two individual normal distribution functions intersect. If no intersection was found between the means of the two normal distribution functions, the mid-point of the means was used as a threshold.

### Computing replication scores, RT values and variability scores

Genome-wide replication score was defined as the percentage of replicated genomic bins for each cell. Throughout the manuscript we have used a 50 kb bin size, but we obtained similar results when using 25 and 100 kb bin size. Cells with a replication score greater than 90% and less than 10% were excluded from downstream analyses. We used the replication score to rank cells by S-phase progression for visualization of their replication status on heatmaps (Fig. 1c). Next we calculated raw RT values as the fraction of cells that replicated the given genomic bin for each stage, respectively. A RT value indicates earlier RT, because a higher proportion of cells replicated the bin. To correct for potential sampling bias of cells, we calculated the fraction of replicated cells in overlapping intervals of the genome-wide replication score with interval size of 35% and increment of 4.33% (for example, 0–35%, 4.33–39.33% and so on) for each genomic bin. The average of these 16 intervals served as the interval RT value that was used for both visualization of RT profiles (Fig. 1e) and downstream analyses. Raw and interval-averaged RT values looked similar overall (Extended Data Fig. 1c; RT raw versus interval), except for some stages in which the number of cells within replication score intervals showed a different distribution. Variability score was calculated using the following formula: score = 1 − (abs(*p* − 0.5)/0.5), where *p* is the fraction of replicated cells (ones) for the given bin; note that *p* is corrected for sampling (as described above). The variability score is therefore a measure of variation in the RT programme across cells, because it represents the number of cells that either replicated or did not replicate a given bin. A value of 1 means that one-half of the cells replicated a given bin and corresponds to the highest variance; likewise, a value of 0 means that either all cells replicated or did not replicate a given bin, which corresponds to the lowest variance and/or no variance.

### Identification of initiation zones (referred to as RT peaks), TTRs and termination zones (referred to as RT troughs)

To distinguish the features of RT, initiation zones, TTRs and termination zones were defined based on RT values. Genomic bins were grouped into 15 clusters by their RT values using the Mclust function from the R package mclust (v.5.4.10, R v.4.1.2). Clusters were ranked by their average RT values following analysis similar to that described previously^10^, except that we used RT values for clustering as opposed to the 16 Repli-seq fractions. Initiation zones and termination zones were defined as consecutive bins with local maxima or minima of their cluster ranks, respectively, in sliding windows of 21 genomic bins using the rollappy function from the R package zoo (v.1.8-10). Regions between initiation zones and termination zones were defined as TTRs (Extended Data Fig. 3b). The number of initiation zones, which we refer to as RT peaks, recorded previously^10^ (approximtely 2,200 in neuronal progenitor cells) is similar to that reported here. To determine the significance of the changes in the number or region size of initiation zones, TTRs and termination zones throughout development, a linear model was fitted using the lm function in R (v.4.1.2). The rank of the developmental stages (that is, 1–7) served as the independent variable. The dependent variable was either the number of regions or the upper quartile of region sizes (75th percentile) for each region type. The *P* value of the coefficient corresponding to the slope indicates the significance of the linear trend. For composite plots, RT values were centred at the middle point of RT peak coordinates in 2 Mb windows and the median of RT values was calculated per position (Fig. 1h). To visualize relative RT compared with the neighbouring region, the minimum value of the 2 Mb window was subtracted for each stage.

### Analysis of RT heterogeneity

Heterogeneity analysis was performed using the sigmoidal model formula as described previously^5,63^. A sigmoidal curve was fitted for each genomic bin by the nls function from the R package stats (v.4.1.2), such that nls(*y* ~ 100/(1 + exp(−*g* × (*x* − *M*))), start = list(*g* = 0.1, *M* = *m*0)) (Extended Data Fig. 6a). The average genome-wide replication score of each of the 16 overlapping intervals (see above) served as the independent variable (*x*), with the percentage of cells that replicated the bin within the same replication score interval as dependent variable (*y*). Model parameters were *M* = mid-point, *g* = slope (gain) and *m*0 = initial value for *M* (100 minus the mean of *y* values). By this method, the replication status of the given genomic bin was related to the overall S-phase progression of cells (measured in intervals of replication score). To anchor the start and end points of the curve, 16 data points of 0 and 100 values were added to the *x* and *y* variable, respectively. Two parameters were calculated from the curve fitting, *M*-value and *T*_width_. The *M*-value (RT mid-point, sometimes also referred to as *T*_rep_ in the literature^10^) is the replication score (roughly S-phase time) at which 50% of the cells replicated the given bin. A higher *M*-value indicates later RT. *T*_width_ is a measure of RT heterogeneity and is defined as the replication score difference (approximate S-phase time difference) of between 25 and 75% of the cells that replicated the given genomic bin. A higher *T*_width_ value indicates higher heterogeneity, because the transition from non-replicated to replicated status is greater.

### Allele-specific analyses

To address any bias that could have been caused by SNPs during alignment, reads were realigned to a SNP-masked genome sequence containing an ‘N’ anywhere in which a SNP between any of the paternal (DBA) or maternal genomes (C57BL/6 × CBA) is located. The bam files were subsequently divided into paternal and maternal reads. Importantly, not all potential SNPs between strains were used. Splitting considered only SNPs that were different for the three genomes or those whose nucleotide was the same for both maternal genomes but different compared with the paternal one. Both reference preparation and splitting were performed with SNPsplit^64^ (v.0.5.0). Reads were filtered using the same tools and thresholds as described above for non-allelic analyses—that is, taking into account read duplication, properly paired criteria and a mapping quality filter. Finally, as previously described, BEDtools intersect was used to count the number of reads for each contiguous 50 kb window. All subsequent analyses were performed on genomic bins, with at least five reads assigned either to the maternal or paternal genome of the same sample.

To determine allelic bias, the log_2_ ratio of maternal:paternal read counts was calculated for each bin. The majority of physically separated maternal or paternal pronuclei showed a high positive (over +2) or negative (below −2) log_2_ ratio, respectively. Pronuclei with a log_2_ ratio of the opposite sign were exchanged for downstream analyses. We identified several parthenogenic examples among IVF zygotes (log_2_ ratio above 1), which were excluded from further analyses. Finally we calculated Spearman’s correlation coefficients on log_2_ maternal:paternal ratios pairwise across single zygotes and visualized these as a correlation heatmap (Extended Data Fig. 4f). A high correlation value between two zygotes indicates that, if a genomic bin has a high allelic bias in one of the zygotes it also has a high bias in the other.

### Analysis of imprinted genes

Lists of maternally and paternally imprinted genes were downloaded from the Geneimprint database (https://www.geneimprint.com/site/genes-by-species.Mus+musculus). RT values were extracted for genomic bins overlapping imprinted genes. If multiple bins overlapped the same gene, RT values were averaged. For expression level and allelic bias analysis, supplementary data were downloaded from Gene Expression Omnibus (GEO) (GSE38495 and GSE45719)^65^. A gene was considered expressed when its average fragments per kilobase exon per million mapped reads value in the given stage was greater than zero. Allelic bias was calculated as the log_2_-transfomed ratio between read counts assigned to Cast or C57BL/6 genomes. A gene was considered maternally biased if the average log^2^ allelic ratio was greater than zero, and paternally biased if less than zero. RT values at imprinted genes were visualized on heatmaps and ordered by their expression and allelic bias status. In total we analysed 49 maternally and 37 paternally imprinted genes, corresponding to 98 and 100 genomic bins, respectively.

### Analysis of transposable elements

Transposable element annotation for the mm10 genome was obtained from Hammell’s laboratory repository (https://labshare.cshl.edu/shares/mhammelllab/www-data/TEtranscripts/TE_GTF/mm10_rmsk_TE.gtf.gz).

Enrichment of transposable elements in RT peaks, TTRs or RT troughs was estimated by calculating the log_2_ ratio of the number of transposable elements of the given type overlapping with RT peaks, TTRs or RT troughs relative to the overlap of randomly shifted transposable elements with RT peaks, TTRs or RT troughs, respectively. The final enrichment value was the average of 1,000 iterations.

### Statistical and genome-wide enrichment analysis

For statistical analyses of single-cell RT data we established a bootstrapping approach and calculated 95% confidence intervals to judge statistical significance^66^. We chose this method to avoid the inflation of *P* values when *n* is large due to a large number of genomic bins (*n* = approximately 49,000) and thus we applied bootstrapping to samples, in this case single cells (*n* = approximately 30–70), rather than to genomic bins. Namely, we iteratively resampled individual cells with replacement 1,000 times for each stage or condition. For each iteration we recalculated RT values and any subsequent statistic—for example, Spearman’s correlation coefficient or ΔRT between conditions, as described above. We constructed confidence intervals from the bootstrap distribution using the percentile method. The 95% confidence interval is the interval between the 2.5th and 97.5th percentiles of the distribution; when 95% confidence intervals do not include zero or two intervals do not overlap, they are significantly different from zero or different from each other, respectively. For enrichment analysis of overlapping regions or gene classes, genomic bins were grouped by significantly differential RT values to increasing (earlier), decreasing (later) or non-significant (no change) bins. Enrichments were visualized on heatmaps by calculating the ratio of the observed number of overlapping bins relative to the expected value, which is the product of the row and column sums divided by the total number of bins in the corresponding contingency table.

### Analysis of public chromatin datasets

Published datasets were downloaded from GEO with accession numbers GSE66581, GSE101571 (ATAC-seq^36^), GSE71434 (H3K4me3 chromatin immunoprecipitation sequencing (ChIP)^34^), GSE112834 (H3K36me3 ChIP^67^), GSE98149 (H3K9me3 ChIP^68^), GSE73952 (H3K27me3ChIP^39^) GSE76687 (H3K27me3 ChIP^69^) and GSE135457 (Pol2 Stacc-seq^52^) andGSE76642 (DNase I hypersensitive sites sequencing^70^). Paired-end reads were trimmed by cutadapt (v.3.4) with parameters -a CTGTCTCTTATA -A CTGTCTCTTATA -a AGATCGGAAGAGC -A AGATCGGAAGAGC --minimum-length=20. Following trimming, reads were aligned to the mouse reference (GRCm38) using bowtie2 (v.2.3.5) with parameters --end-to-end --very-sensitive --no-unal --no-mixed --no-discordant -I 10 -X 500. Reads were filtered by mapping quality score using SAMtools (v.1.3) with the parameter -q 12. Read pairs were read into R (v.3.6.3) using the readGAlignmentPairs function from the GenomicAlignment package (v.1.22.0) and were filtered for unique fragments. Fragments aligned to the mitochondrial genome or small scaffolds were not considered in analyses. Fragments were counted in 50 kb consecutive genomic bins (same bins as for RT profiles), normalized by the sum of fragment counts and multiplied by 1 million. Finally, normalized counts were log_2_ transformed following the addition of a pseudocount of 1. Note that, for the analysis of H3K27me3 in Extended Data Fig. 10b,c the dataset used was that of Liu et al. (GSE73952)^39^ whereas in Fig. 5f the dataset used was that of Zheng et al.^69^ (GSE76687). For the correlation analysis shown in Fig. 5f we used the following stages when the actual stage was not available: early 2-cell ATAC-seq for zygote, morula DNase I hypersensitive sites sequencing for ICM and ES cell LmnB1 DamID for ICM. Differential genomic bins between conditions (for example, ATAC-seq following α-amanitin treatment) were called by DESeq2 (v.1.34.0) with an adjusted *P* value cutoff of 0.05. For ATAC-seq analysis in α-amanitin-treated embryos, 2-cell-stage embryos administered α-amanitin treatment by Wu et al.^37^ (GSE101571) were compared with untreated 2-cell-stage embryos derived from Wu et al.^36^ (GSE66581).

### Analysis of public HiC and LAD datasets

HiC compartment coordinates and scores (GSE82185)^16^, as well as LAD coordinates (GSE112551)^13^, were analysed as previously described^13^.

### Reporting summary

Further information on research design is available in the Nature Portfolio Reporting Summary linked to this article.

## Online content

Any methods, additional references, Nature Portfolio reporting summaries, source data, extended data, supplementary information, acknowledgements, peer review information; details of author contributions and competing interests; and statements of data and code availability are available at 10.1038/s41586-023-06872-1.

### Supplementary information

Reporting Summary

Supplementary Table 1 Metrics and quality control data of single-cell Repli-seq samples. Overview of sample collection, mapping statistics and quality control. Cells with a high coefficient of variation were removed from analyses and the coefficient of variation was recalculated, excluding each chromosome separately. Cells were considered for further analysis if they passed the threshold when only one specific chromosome was removed.

## Data Availability

The scRepli-seq data for the present study are available from the GEO database, accession GSE218365. Previously published RNA sequencing datasets reanalysed in the present study are available under accessions GSE38495, GSE45719 and GSE98063. Chromatin datasets reanalysed in the present study are available under accessions. GSE66581, GSE101571, GSE71434, GSE112834, GSE98149, GSE73952, GSE76687, GSE135457 and GSE76642. All other data supporting the findings of the present study are available from the corresponding author on reasonable request. HiC and LAD datasets reanalysed in the present study are available under accessions GSE82185 and GSE112551.

## Code availability

Next-generation sequencing data were analysed with publicly available programmes and packages, as detailed in Methods. Additional details on specific code used to generate scRepli-seq workflows are available on request.

## References

[1] Emerson DJ (2022). Cohesin-mediated loop anchors confine the locations of human replication origins. Nature.

[2] Pope BD (2014). Topologically associating domains are stable units of replication-timing regulation. Nature.

[3] Ryba T (2011). Replication timing: a fingerprint for cell identity and pluripotency. PLoS Comput. Biol..

[4] Klein KN (2021). Replication timing maintains the global epigenetic state in human cells. Science.

[5] Dileep V, Gilbert DM (2018). Single-cell replication profiling to measure stochastic variation in mammalian replication timing. Nat. Commun..

[6] Gilbert, D. M. & Gasser, S. M. in *DNA Replication and Human Disease* (ed. DePamphilis, M. L.) (Cold Spring Harbor Laboratory Press, 2006).

[7] Hiratani I (2008). Global reorganization of replication domains during embryonic stem cell differentiation. PLoS Biol..

[8] Fragkos M, Ganier O, Coulombe P, Mechali M (2015). DNA replication origin activation in space and time. Nat. Rev. Mol. Cell Biol..

[9] Farkash-Amar S (2008). Global organization of replication time zones of the mouse genome. Genome Res..

[10] Zhao PA, Sasaki T, Gilbert DM (2020). High-resolution Repli-Seq defines the temporal choreography of initiation, elongation and termination of replication in mammalian cells. Genome Biol..

[11] Petryk N (2016). Replication landscape of the human genome. Nat. Commun..

[12] Burton A, Torres-Padilla ME (2014). Chromatin dynamics in the regulation of cell fate allocation during early embryogenesis. Nat. Rev. Mol. Cell Biol..

[13] Borsos M (2019). Genome-lamina interactions are established de novo in the early mouse embryo. Nature.

[14] Ke Y (2017). 3D chromatin structures of mature gametes and structural reprogramming during mammalian embryogenesis. Cell.

[15] Flyamer IM (2017). Single-nucleus Hi-C reveals unique chromatin reorganization at oocyte-to-zygote transition. Nature.

[16] Du Z (2017). Allelic reprogramming of 3D chromatin architecture during early mammalian development. Nature.

[17] Schultz RM (1993). Regulation of zygotic gene activation in the mouse. Bioessays.

[18] Seller CA, O’Farrell PH (2018). Rif1 prolongs the embryonic S phase at the *Drosophila* mid-blastula transition. PLoS Biol..

[19] Bartlett DA, Dileep V, Baslan T, Gilbert DM (2022). Mapping replication timing in single mammalian cells. Curr. Protoc..

[20] Chazaud C, Yamanaka Y, Pawson T, Rossant J (2006). Early lineage segregation between epiblast and primitive endoderm in mouse blastocysts through the Grb2-MAPK pathway. Dev. Cell.

[21] Streffer C, van Beuningen D, Molls M, Zamboglou N, Schulz S (1980). Kinetics of cell proliferation in the pre-implanted mouse embryo in vivo and in vitro. Cell Tissue Kinet..

[22] Tubbs A (2018). Dual roles of Poly(dA:dT) tracts in replication initiation and fork collapse. Cell.

[23] Petryk N (2018). MCM2 promotes symmetric inheritance of modified histones during DNA replication. Science.

[24] Dileep V, Rivera-Mulia JC, Sima J, Gilbert DM (2015). Large-scale chromatin structure-function relationships during the cell cycle and development: insights from replication timing. Cold Spring Harb. Symp. Quant. Biol..

[25] Miura H (2019). Single-cell DNA replication profiling identifies spatiotemporal developmental dynamics of chromosome organization. Nat. Genet..

[26] Bouniol-Baly C, Nguyen E, Besombes D, Debey P (1997). Dynamic organization of DNA replication in one-cell mouse embryos: relationship to transcriptional activation. Exp. Cell. Res..

[27] Barton SC (2001). Genome-wide methylation patterns in normal and uniparental early mouse embryos. Hum. Mol. Genet..

[28] Rivera-Mulia JC (2018). Allele-specific control of replication timing and genome organization during development. Genome Res..

[29] Du Q (2021). DNA methylation is required to maintain both DNA replication timing precision and 3D genome organization integrity. Cell Rep..

[30] Yang SC, Rhind N, Bechhoefer J (2010). Modeling genome-wide replication kinetics reveals a mechanism for regulation of replication timing. Mol. Syst. Biol..

[31] Kupper K (2007). Radial chromatin positioning is shaped by local gene density, not by gene expression. Chromosoma.

[32] Abe K, Schauer T, Torres-Padilla ME (2022). Distinct patterns of RNA polymerase II and transcriptional elongation characterize mammalian genome activation. Cell Rep..

[33] Dahl JA (2016). Broad histone H3K4me3 domains in mouse oocytes modulate maternal-to-zygotic transition. Nature.

[34] Zhang B (2016). Allelic reprogramming of the histone modification H3K4me3 in early mammalian development. Nature.

[35] Bensaude O (2011). Inhibiting eukaryotic transcription: which compound to choose? How to evaluate its activity?. Transcription.

[36] Wu J (2016). The landscape of accessible chromatin in mammalian preimplantation embryos. Nature.

[37] Wu J (2018). Chromatin analysis in human early development reveals epigenetic transition during ZGA. Nature.

[38] Burton A (2020). Heterochromatin establishment during early mammalian development is regulated by pericentromeric RNA and characterized by non-repressive H3K9me3. Nat. Cell Biol..

[39] Liu X (2016). Distinct features of H3K4me3 and H3K27me3 chromatin domains in pre-implantation embryos. Nature.

[40] Dixon JR (2012). Topological domains in mammalian genomes identified by analysis of chromatin interactions. Nature.

[41] Lu JY (2021). Homotypic clustering of L1 and B1/Alu repeats compartmentalizes the 3D genome. Cell Res..

[42] Wijchers PJ (2015). Characterization and dynamics of pericentromere-associated domains in mice. Genome Res..

[43] Fadloun A (2013). Chromatin signatures and retrotransposon profiling in mouse embryos reveal regulation of LINE-1 by RNA. Nat. Struct. Mol. Biol..

[44] Peaston AE (2004). Retrotransposons regulate host genes in mouse oocytes and preimplantation embryos. Dev. Cell.

[45] Jachowicz JW (2017). LINE-1 activation after fertilization regulates global chromatin accessibility in the early mouse embryo. Nat. Genet..

[46] Meuleman W (2013). Constitutive nuclear lamina-genome interactions are highly conserved and associated with A/T-rich sequence. Genome Res..

[47] Evsikov AV (2004). Systems biology of the 2-cell mouse embryo. Cytogenet. Genome Res..

[48] Nakatani T (2022). DNA replication fork speed underlies cell fate changes and promotes reprogramming. Nat. Genet..

[49] Peric-Hupkes D (2010). Molecular maps of the reorganization of genome-nuclear lamina interactions during differentiation. Mol. Cell.

[50] Ishiuchi T (2021). Reprogramming of the histone H3.3 landscape in the early mouse embryo. Nat. Struct. Mol. Biol..

[51] Zhang S (2021). RNA polymerase II is required for spatial chromatin reorganization following exit from mitosis. Sci. Adv..

[52] Liu B (2020). The landscape of RNA Pol II binding reveals a stepwise transition during ZGA. Nature.

[53] Smith RK, Johnson MH (1986). Analysis of the third and fourth cell cycles of mouse early development. J. Reprod. Fertil..

[54] Park SJ, Shirahige K, Ohsugi M, Nakai K (2015). DBTMEE: a database of transcriptome in mouse early embryos. Nucleic Acids Res..

[55] Aoki F, Worrad DM, Schultz RM (1997). Regulation of transcriptional activity during the first and second cell cycles in the preimplantation mouse embryo. Dev. Biol..

[56] Goddard MJ, Pratt HP (1983). Control of events during early cleavage of the mouse embryo: an analysis of the ‘2-cell block’. J. Embryol. Exp. Morphol..

[57] Burton A (2013). Single-cell profiling of epigenetic modifiers identifies PRDM14 as an inducer of cell fate in the mammalian embryo. Cell Rep..

[58] Langmead B, Salzberg SL (2012). Fast gapped-read alignment with Bowtie 2. Nat. Methods.

[59] Danecek P (2021). Twelve years of SAMtools and BCFtools. Gigascience.

[60] Quinlan AR, Hall IM (2010). BEDTools: a flexible suite of utilities for comparing genomic features. Bioinformatics.

[61] Nilsen G (2012). Copynumber: efficient algorithms for single- and multi-track copy number segmentation. BMC Genomics.

[62] Benaglia, T. et al. Mixtools: an R package for analyzing finite mixture models. *J. Stat. Softw*. jstatsoft.org/article/view/v032i06 (2009).

[63] Takahashi S (2019). Genome-wide stability of the DNA replication program in single mammalian cells. Nat. Genet..

[64] Krueger F, Andrews SR (2016). SNPsplit: allele-specific splitting of alignments between genomes with known SNP genotypes. F1000Res..

[65] Deng Q, Ramskold D, Reinius B, Sandberg R (2014). Single-cell RNA-seq reveals dynamic, random monoallelic gene expression in mammalian cells. Science.

[66] Efron, B. The Jackknife, the Bootstrap and other resampling plans. In *CBMS-NSF Regional Conference Series in Applied Mathematics* (1982); 10.1137/1.9781611970319.

[67] Xu Q (2019). SETD2 regulates the maternal epigenome, genomic imprinting and embryonic development. Nat. Genet..

[68] Wang C (2018). Reprogramming of H3K9me3-dependent heterochromatin during mammalian embryo development. Nat. Cell Biol..

[69] Zheng H (2016). Resetting epigenetic memory by reprogramming of histone modifications in mammals. Mol. Cell.

[70] Lu F (2016). Establishing chromatin regulatory landscape during mouse preimplantation development. Cell.

