# Supplementary material for: Emergence of replication timing during early mammalian development
Source: Nature. 2023 Dec 20;625(7994):401–9. doi: 10.1038/s41586-023-06872-1 (PMC10781638; doi:10.1038/s41586-023-06872-1)
Supplement: Supplementary file 1 — Reporting Summary [file 41586_2023_6872_MOESM1_ESM.pdf]

nature portfolio

Corresponding author(s): Maria-Elena Torres-Padilla

Last updated by author(s): Oct 12, 2023

Reporting Summary

Nature Portfolio wishes to improve the reproducibility of the work that we publish. This form provides structure for consistency and transparency in reporting. For further information on Nature Portfolio policies, see our Editorial Policies and the Editorial Policy Checklist.

Statistics

For all statistical analyses, confirm that the following items are present in the figure legend, table legend, main text, or Methods section.

n/a	Confirmed	
☐	☒	The exact sample size (*n*) for each experimental group/condition, given as a discrete number and unit of measurement
☐	☒	A statement on whether measurements were taken from distinct samples or whether the same sample was measured repeatedly
☐	☒	The statistical test(s) used AND whether they are one- or two-sided<br>*Only common tests should be described solely by name; describe more complex techniques in the Methods section.*
☒	☐	A description of all covariates tested
☐	☒	A description of any assumptions or corrections, such as tests of normality and adjustment for multiple comparisons
☐	☒	A full description of the statistical parameters including central tendency (e.g. means) or other basic estimates (e.g. regression coefficient) AND variation (e.g. standard deviation) or associated estimates of uncertainty (e.g. confidence intervals)
☐	☒	For null hypothesis testing, the test statistic (e.g. *F*, *t*, *r*) with confidence intervals, effect sizes, degrees of freedom and *P* value noted<br>*Give P values as exact values whenever suitable.*
☒	☐	For Bayesian analysis, information on the choice of priors and Markov chain Monte Carlo settings
☒	☐	For hierarchical and complex designs, identification of the appropriate level for tests and full reporting of outcomes
☒	☐	Estimates of effect sizes (e.g. Cohen's *d*, Pearson's *r*), indicating how they were calculated

Our web collection on statistics for biologists contains articles on many of the points above.

Software and code

Policy information about availability of computer code

Data collection	LAS X (version 3) was used for imaging data acquisition and ImageJ (version 1.53k) for image processing.
Data analysis	The following software were used for scRepli-seq data processing and analysis (see details in Methods): bowtie2 (version 2.3.5), samtools (version 1.3 and 1.9), bedtools (version 2.29.0), R (version 3.6.3, 4.0.0,and 4.1.2), R package copynumber (version 1.28.0), R package mixtools (version 1.2.0), R package mclust (version 5.4.10), R package zoo (version 1.8-10), GenomicAlignment package (version 1.22.0), R lm and nls functions (version 4.1.2) and SNPsplit (version 0.5.0).<br>Custom code is available upon request.<br>Adobe Illustrator CS6 (version 16) and Adobe Photoshop CS6 (version 13) were used for Figure preparation.<br>Ct value for qPCR was determined by LightCycler® 96 Software (version 1.1.0.1320).

For manuscripts utilizing custom algorithms or software that are central to the research but not yet described in published literature, software must be made available to editors and reviewers. We strongly encourage code deposition in a community repository (e.g. GitHub). See the Nature Portfolio guidelines for submitting code & software for further information.

Data

Policy information about availability of data

All manuscripts must include a data availability statement. This statement should provide the following information, where applicable:

- Accession codes, unique identifiers, or web links for publicly available datasets
- A description of any restrictions on data availability
- For clinical datasets or third party data, please ensure that the statement adheres to our policy

The scRepli-seq data from this study are available from the Gene Expression Omnibus, accession number GSE218365.

Published datasets were downloaded from GEO with accession numbers GSE101571 and GSE66581 (ATAC-seq), GSE38495, GSE45719 and GSE98063 (RNA-seq), GSE71434 (H3K4me3 ChIP), GSE112834 (H3K36me3 ChIP), GSE98149 (H3K9me3 ChIP), GSE76687 and GSE73952 (H3K27me3 ChIP), GSE82185 (Hi-C), GSE135457 (Pol2 Stacc-seq), GSE76642 (DNase I-seq) and GSE112551 (lamin B1 DamID).
For expression level and allelic bias analysis supplementary data were downloaded from Gene Expression Omnibus (GSE38495 and GSE45719).
TE annotation for the mm10 genome was obtained from the Hammell´s lab repository (https://labshare.cshl.edu/shares/mhammelllab/www-data/TEtranscripts/TE_GTF/mm10_rmsk_TE.gtf.gz).
Gene classes (e.g. maternal RNA or major ZGA) were obtained from the DBTMEE database (https://dbtmee.hgc.jp).

Human research participants

Policy information about studies involving human research participants and Sex and Gender in Research.

Reporting on sex and gender	N/A
Population characteristics	N/A
Recruitment	N/A
Ethics oversight	N/A

Note that full information on the approval of the study protocol must also be provided in the manuscript.

Field-specific reporting

Please select the one below that is the best fit for your research. If you are not sure, read the appropriate sections before making your selection.

☒ Life sciences ☐ Behavioural & social sciences ☐ Ecological, evolutionary & environmental sciences

For a reference copy of the document with all sections, see nature.com/documents/nr-reporting-summary-flat.pdf

Life sciences study design

All studies must disclose on these points even when the disclosure is negative.

Sample size	Sample size was chosen in order to ensure that the data was consistent and reproducible. To do the statistical test, at least 3 biological replicates were included based on previously published work and preliminary studies as standard for this field of research.<br>See Figure legends for each experiment.
Data exclusions	No data were excluded.
Replication	EU and EdU data was replicated at least twice; for the Repli-seq analyses, sample collection was done at least twice on each condition and the total number of cells is indicated in each figure panel. For generating Repli-seq data, sample collection was at least twice on each stages or conditions. All attempts at replication were successful as reported in the manuscript.
Randomization	Cells and embryos were allocated at random to experimental groups as stated in the Methods
Blinding	No experiment presented a subjective data collection that would require blinding. Experimentors were not blinded during experimental group allocation because no subjective data collection was done in this work.

Reporting for specific materials, systems and methods

We require information from authors about some types of materials, experimental systems and methods used in many studies. Here, indicate whether each material, system or method listed is relevant to your study. If you are not sure if a list item applies to your research, read the appropriate section before selecting a response.

Materials & experimental systems

n/a	Involved in the study
☐	☒ Antibodies
☒	☐ Eukaryotic cell lines
☒	☐ Palaeontology and archaeology
☐	☒ Animals and other organisms
☒	☐ Clinical data
☒	☐ Dual use research of concern

Methods

n/a	Involved in the study
☒	☐ ChIP-seq
☒	☐ Flow cytometry
☒	☐ MRI-based neuroimaging

Antibodies

Antibodies used	Antibodies used were as follows: anti-RNA polymerase II (sc-899), anti-Pol II Ser2P (ab5095), anti-H3K4me3 (C15410003). Secondary antibody used in this study was Goat anti-Rabbit IgG (H+L) Highly Cross-Adsorbed Secondary Antibody, Alexa Fluor™ 488 (ThermoFisher SCIENTIFIC, A11034, Lot2110499)
Validation	All antibodies were validated by manufacturers (https://www.abcam.com/products/primary-antibodies/rna-polymerase-ii-ctd-repeat-ysptsps-phospho-s2-antibody-ab5095.html) (https://www.google.com/url?sa=t&rct=j&q=&esrc=s&source=web&cd=&cad=rja&uact=8&ved=2ahUKEwj30a7pld-BAxVK3QIHHe8xDIgQFnoECA0QAQ&url=https%3A%2F%2Fdatasheets.scbt.com%2Fsc-899.pdf&usg=AOvVaw2PwLQWUofAKLWoWVBZbOYg&opi=89978449) (https://www.diagenode.com/en/p/h3k4me3-polyclonal-antibody-premium-50-ug-50-ul) and in our previous studies (Abe, Cell Rep., 2022, and Borsos, Nature, 2019).

Animals and other research organisms

Policy information about studies involving animals; ARRIVE guidelines recommended for reporting animal research, and Sex and Gender in Research

Laboratory animals	5-8 weeks old F1 (C57BL6 X CBA/H) mice were used to provide oocytes and crossed with 3-6 months old DBA/2J males to provide zygotes. Housing temperature, humidity, and light cycle of mouse cage are kept , 20-24 degrees celsius, 45-65%, and 12h dark/12h light, respectively.
Wild animals	This study did not use wild animals.
Reporting on sex	Embryos from both sexes were collected randomly, without prior knowledge of sex.
Field-collected samples	This study did not involve field-collected samples.
Ethics oversight	All experiments were performed under the authorization of the Upper Bavarian authorities.

Note that full information on the approval of the study protocol must also be provided in the manuscript.
